# Identification and Comprehensive Analysis of the *Nuclear Factor-Y* Family Genes Reveal Their Multiple Roles in Response to Nutrient Deficiencies in *Brassica napus*

**DOI:** 10.3390/ijms221910354

**Published:** 2021-09-26

**Authors:** Xinrui Zheng, Hao Zhang, Limei Zhang, Fangsen Xu, Lei Shi, Sheliang Wang, Juan Hong, Guangda Ding

**Affiliations:** 1 Key Laboratory of Arable Land Conservation (Middle and Lower Reaches of Yangtze River), Ministry of Agriculture and Rural Affairs, State Environmental Protection Key Laboratory of Soil Health and Green Remediation, Ministry of Ecology and Environment, Microelement Research Center, College of Resources and Environment, Huazhong Agricultural University, Wuhan 430070, China; zxr414@webmail.hzau.edu.cn (X.Z.); hao.zhang@webmail.hzau.edu.cn (H.Z.); lmzhang@mail.hzau.edu.cn (L.Z.); fangsenxu@mail.hzau.edu.cn (F.X.); leish@mail.hzau.edu.cn (L.S.); sheliangwang2017@mail.hzau.edu.cn (S.W.); 2 Institute of Environment and Safety, Wuhan Academy of Agricultural Sciences, Wuhan 430070, China

**Keywords:** *Brassica napus*, *Nuclear Factor-Y*, transcription factor, genome-wide characterization, nutrient deficiency response, expression regulation

## Abstract

*Nuclear Factor-Y* (*NF-Y*) transcription factors play vital roles in plant abiotic stress response. Here, the *NF-Y* family in *Brassica napus*, which is hyper-sensitive to nitrogen (N) deprivation, was comprehensively identified and systematically characterized. A total of 108 *NF-Y* family members were identified in *B. napus* and categorized into three subfamilies (38 *NF-YA*, 46 *NF-YB* and 24 *NF-YC*; part of the *Arabidopsis NF-YC* homologous genes had been lost during *B. napus* evolution). In addition, the expansion of the *NF-Y* family in *B. napus* was driven by whole-genome duplication and segmental duplication. Differed expression patterns of *BnaNF-Ys* were observed in response to multiple nutrient starvations. Thirty-four genes were regulated only in one nutrient deficient condition. Moreover, more *BnaNF-YA* genes were differentially expressed under nutrient limited environments compared to the *BnaNF-YB* and *BnaNF-YC* subfamilies. Sixteen hub genes responded diversely to N deprivation in five rapeseed tissues. In summary, our results laid a theoretical foundation for the follow-up functional study of the key *NF-Y* genes in *B. napus* in regulating nutrient homeostasis, especially N.

## 1. Introduction

*Nuclear Factor-Y* (*NF-Y*) transcription factors were discovered in yeast, mammals, plants, and other eukaryotes [1,2,3]. The *NF-Y* family members can specifically recognize and bind to the CCAAT sequence in the target gene’s promoters [4]. The *NF-Y* transcription factor complex is composed of protein subunits from three unique subfamilies: *NF-YA*, *NF-YB* and *NF-YC*. A comparison of the protein sequences of three *NF-Y* subunits in different species, reveals that each subunit contains a conserved structural region. The *NF-YA* subunit has a carbon terminal region containing 56 amino acids, the *NF-YB* subunit a middle region containing 90 amino acids, and the *NF-YC* subunit a nitrogen (N) terminal region encompassing 84 amino acids [3]. The *NF-Y* family has been functionally annotated in a number of species. In yeast and animals, all *NF-Y* subunits contain only one or two members, but in plants, the number of genes encoding *NF-Y* subunits is expanded significantly [2]. In *Arabidopsis thaliana*, there are 36 *NF-Y* genes including 10, 13 and 13 members in the *NF-YA*, *NF-YB*, and *NF-YC* subunits, respectively. In theory, these subunits can be integrated into 1690 distinct complexes [5]. Likewise, in rice (*Oryza sativa*), 10, 11, and 7 members were identified in the *NF-YA*, *NF-YB*, and *NF-YC* subfamilies, respectively [6]. To date, the number of identified members of the three subfamilies in other crop plants have previously been studied, such as soybean [7], tomato [8], maize [9], sorghum [10,11], citrus [12], barley [13], cucumber [14], and apple [15]. The results indicate that the numbers of *NF-Y* family members have increased in plants. Therefore, these *NF-Y* family genes may combine with each other to form tens of thousands of potential heterotrimers in plants [16]. The differentiation of the *NF-Y* family genes may lead to the diversity of their functions.

In addition to regulating a wide range of growth and development processes, several *NF-Y* subfamily members also play crucial roles in plant abiotic stress tolerance [4]. Studies in plants have shown that *NF-Y* transcription factors are involved in the regulation of seed germination [17], flowering time [18,19,20], root elongation [21], fruit ripening [8], embryogenesis [22], photosynthesis [23], chloroplast biogenesis [24], and fatty acid biosynthesis [25]. For example, several *NF-Y* proteins interact with photoperiod-induced flowering pathway regulators in *Arabidopsis* and tomato, and overexpression of the *NF-YA1* and *NF-YB1* genes in *Arabidopsis* lead to delayed flowering in plants [18,19]. In addition to plant growth regulation, *NF-Ys* are widely involved in abiotic stress responses, such as osmotic stresses caused by drought and salt, via ABA-dependent or independent pathways [2,4,26]. For instance, the expression levels of most of the *NF-Y* genes in *Arabidopsis* increase under drought stress condition [27]. Overexpression of *AtNF-YB1*, *AtNF-YB2* and *AtNF-YB5* significantly improves *Arabidopsis* drought tolerance by mediating the expression of stress-responsive genes [28,29,30]. In rice, drought stress induces the expression of *OsNF-YA7* which enhances plant drought tolerance through an ABA-independent manner [31]. Yet, *TaNF-YB3* was reported to be crucial for plant drought tolerance by modulating an ABA-associated signaling pathway [32].

Furthermore, *NF-Y* genes act as key players in improving plant adaptability to nutritional constraints [33,34]. It is reported that N and phosphorus (P) deficiencies strongly induce the expression of five *Arabidopsis NF-YA* subfamily members, and N-starvation-induced senescence is significantly alleviated in plants overexpressing *NF-YA* genes [35]. Additionally, *miR169*, which regulates *NF-YA* genes at the post transcriptional level in *Arabidopsis*, is involved in N-starvation response [29,36]. The expression levels of most *NF-YA* genes in wheat are enhanced under both N and P deficiencies, and overexpression of *TaNFYA-B1* stimulates root development as well as the up-regulation of transporters involved in root nitrate and phosphate uptake [37]. These results indicate that *NF-YA* genes can promote wheat to absorb phosphate under low P stress. However, the underlying mechanisms of how *NF-Y* genes regulate nutrient use efficiency are still unknown. Oilseed rape (*Brassica napus*) has been widely planted and harvested to produce vegetable oil, livestock feed and biodiesel [38]. *B. napus* (AACC genome, 2n = 38) is a heterotetraploid crop obtained by crossing *B. oleracea* (CC genome, 2n = 18) and *B. rapa* (AA genome, 2n = 20) [39]. Many duplicated segments and homeologous regions within the genome are generated by the allopolyploidy events in *B. napus* [40], which results in a significant increase in gene copy number variation. It is generally believed that *B. napus* is susceptible to multiple abiotic stresses [38]. *NF-Y* transcription factors have been identified in various plant species [7,8,9,10,12,13,41]. However, little is known about how *NF-Y* genes mediate nutrient stress responses in *B. napus*. Here, the physiological responses of rapeseed to nutrient starvations were investigated, and 108 *B. napus NF-Y* family members were identified using BLASTP search against the protein sequences of *B. napus*. Gene structure, chromosomal location, phylogenetic analysis, conserved motifs, evolutionary patterns and synteny analysis were conducted to characterize the *BnaNF-Y* family members. Moreover, the expression changes of the *BnaNF-Y* genes in response to multiple nutritional deficiencies were analyzed, as were as gene coexpression networks and gene subcellular localization. Collectively, our investigation laid a key foundation for further functional studies of the *NF-Y* family of genes in oilseed rape.

## 2. Results

### 2.1. Physiological Response of B. napus to N, P and K Deficiencies

**Figure 1:**
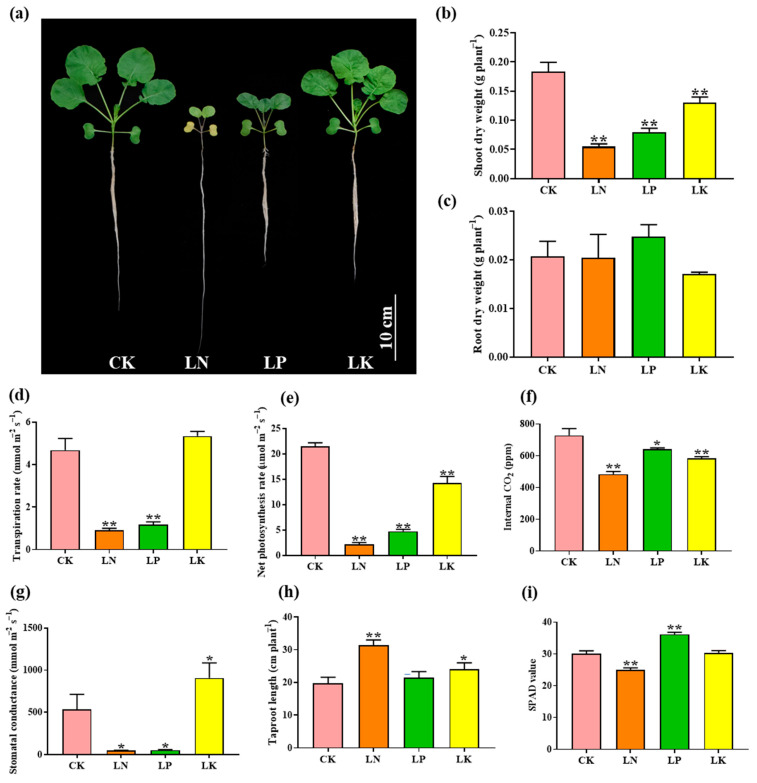
The physiological responses of *B. napus* to N, P and K deficiencies. (**a**) The phenotypes of *B. napus* under N, P and K deficiencies; (**b**) Shoot dry weight; (**c**) Root dry weight; (**d**) Transpiration rate; (**e**) Net photosynthesis rate; (**f**) Internal CO_2_; (**g**) Stomatal conductance; (**h**) Taproot length; (**i**) SPAD value. CK: normal nutrient supply; LN: N deficiency (100 μM N); LP: P deficiency (5 μM P); LK: K deficiency (5 μM K). Error line represents variance (*n* = 4), different letters (**a**–**d**) indicate significant differences per Student’s *t* test, * *p* < 0.05, ** *p* < 0.01.

In order to explore the response of *B. napus* to diverse nutrient deficiencies, the *B. napus* cultivar “ZS11” was cultivated for 11 days under low N (LN, 100 μM), low P (LP, 5 μM) and low K (LK, 5 μM) conditions (Figure 1). Compared with normal nutrient supply (CK), the growth of *B. napus* under nutrient limitation was significantly inhibited, especially under N deficient conditions (Figure 1a–c). As expected, shoot dry weight under LN, LP and LK conditions was reduced to 70.11%, 56.40% and 29.01% of that under CK condition, respectively. However, no difference was observed in roots (Figure 1b,c). Moreover, the taproot growth was the most sensitive under LN condition, which was about 59.91% longer than that under CK. The SPAD value of leaves decreased significantly under LN condition, while the opposite trend was observed under LP condition (Figure 1i). We further determined the photosynthetic characters of *B. napus* under the same conditions. Compared with CK, the transpiration rate, net photosynthesis rate, internal CO_2_ and stomatal conductance were notably reduced under LN and LP conditions, indicating that the lack of N and P greatly inhibited *B. napus* photosynthesis (Figure 1d–g). For K deficiency, it led to 33.72% and 19.67% reduction in net photosynthesis rate and internal CO_2_ while the stomatal conductance was significantly increased, by 70.04% compared to CK. However, little effect was observed on the transpiration rate (Figure 1d–g). In general, *B. napus* has diverse responses to different nutrient deficiencies, and is especially sensitive to N deficiency and less affected by LK.

### 2.2. Identification and Molecular Characterization of the NF-Y Family Genes

**Figure 2:**
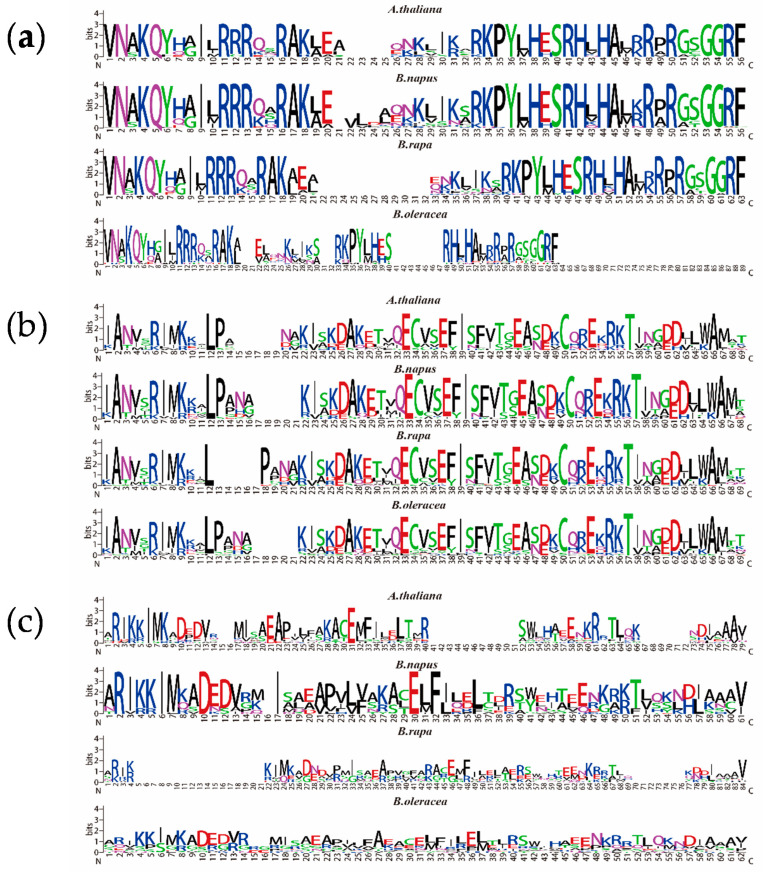
The conserved domains of three *NF-Y* subfamilies of *B. napus*, *B. rapa*, *B. oleracea* and *Arabidopsis*. The logo diagrams illustrate the consensus sequences of conserved domains of the three *NF-Y* subfamilies in different species, which are determined based on the multiple sequence alignments in each species: (**a**) The *Nuclear Factor-YA* (*NF-YA*) subfamily; (**b**) The *Nuclear Factor-YB* (*NF-YB*) subfamily; (**c**) The *Nuclear Factor-YC* (*NF-YC*) subfamily.

Previous reports showed that the *NF-Y* family genes could improve nutrient uptake and assimilation [37]. Thus, in order to explore the role of the *NF-Y* family genes in regulating nutritional deficiency tolerance in *B. napus*, we comprehensively identified the family members. Through multiple BLAST search against the homologue of 36 *Arabidopsis NF-Y* family members, a total of 108 *NF-Y* genes were detected in the whole genome of *B. napus* (Table S1). The *NF-Y* genes of *B. napus* included 38 genes of *NF-YA* subfamily, 46 genes of *NF-YB* subfamily and 24 genes of *NF-YC* subfamily (Tables S2 and S3). Meanwhile, 54 members of the *NF-Y* family (18 *NF-YA*, 24 *NF-YB* and 12 *NF-YC*) in *B. rapa* were identified. In *B. oleracea* 62 (22 *NF-YA*, 27 *NF-YB* and 13 *NF-YC*) genes were identified (Tables S2 and S3). Surprisingly, we found that several copies of the *NF-YC* subfamily members were lost in the whole genomes of three *Brassica* species, including *NF-YC1/3/5/6/7/8/12* (Table S1). We then summarized the *NF-Y* family genes in 14 species. Besides the three *Brassica* species, *Glycine max* has the highest number of *NF-Y* genes, with 68 (21 *NF-YA*, 32 *NF-YB* and 15 *NF-YC*) members, while *Prunus persica* has the least *NF-Y* genes, with 24 (6 *NF-YA*, 12 *NF-YB* and 6 *NF-YC*) members (Table S2). Moreover, multiple sequence alignment was conducted based on the specific conserved protein sequences of three *NF-Y* subfamilies [3]. The results showed that the *NF-YA* and *NF-YB* subfamilies of the three species were relatively conserved, while the *NF-YC* subfamily varied largely, which may be related to the loss of some *NF-YC* subfamily genes during evolution (Figure 2).

**Figure 3:**
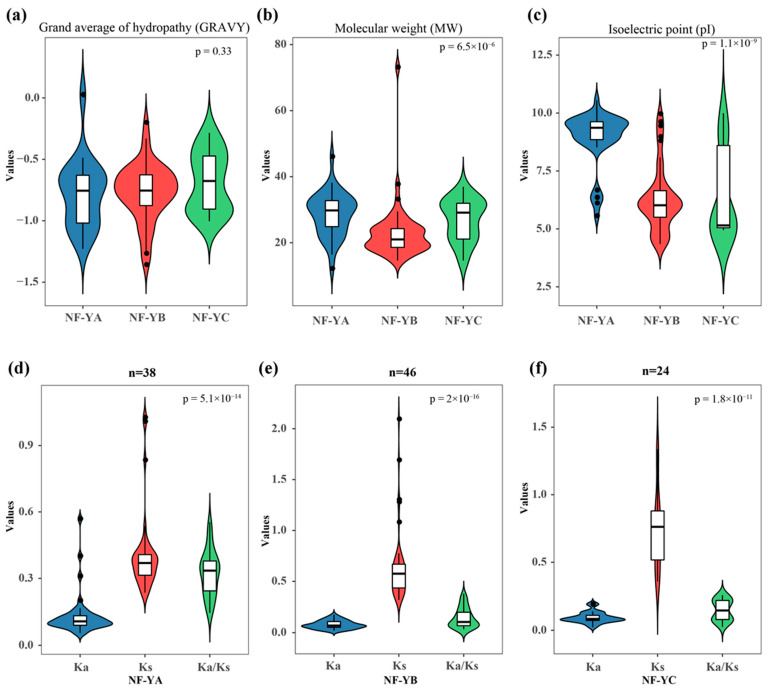
Harp diagrams of the molecular characteristics of *NF-Y* proteins and the synonymous (Ks), nonsynonymous (Ka) and Ka/Ks ratio of each *NF-Y* subfamily in rapeseed. (**a**) Grand average of hydropathy (GRAVY); (**b**) Molecular weight (MW); (**c**) Theoretical isoelectric point (pI); The GRAVY value is defined as the sum of the hydropathy values of the amino acids divided by the protein length. (**d**–**f**) Ka, Ks, Ka/Ks ratio of the *NF-YA*: (**d**) *NF-YB*; (**e**) *NF-YC*; (**f**) Subfamilies.

The gene ID, gene length (coding sequence), gene location, intron number, protein length and predicted subcellular localization of these genes are listed in Table S3. In general, the gene length of *BnaNF-Ys* varied from 324 bp to 2674 bp, and the genes encoding polypeptides varied from 107 to 642 amino acids in length. The intron number of *BnaNF-Ys* ranged from 0 to 7. The subcellular localization predicted by WoLF PSORT indicated that 86 *BnaNF-Y* proteins were localized in nucleus, 17 in chloroplast, two in mitochondria, two in cytoplasm, and one in plasma (Table S3). The grand average of hydropathy (GRAVY) value of the *BnaNF-Y* proteins varied from −1.358 to 0.027, most of them negative except *BnaC06.NF-YA3b* and *BnaC04.NF-YA3*, indicating that most of the *BnaNF-Ys* are hydrophilic (Figure 3a). The molecular weight (MW) of the *BnaNF-Y* proteins varied from 12.211 kDa to 73.177 kDa (Figure 3b), with isoelectronic points (pI) values which ranged from 4.34 to 10.55. The pI of most *NF-YA* subfamily genes was more than seven, but the pI of most *NF-YB* and *NF-YC* subfamily genes were less than seven (Figure 3c).

### 2.3. Phylogenesis of the NF-Y Genes in B. napus

In order to explore the genetic relationships of the *NF-Y* genes between *Arabidopsis* and *B. napus*, the protein sequences of 36 *AtNF-Ys* and 108 *BnaNF-Ys* were used to construct a phylogenetic tree in MEGA7.0 (Figure S1). The results showed that all of the *NF-Y* family members can be divided into three subgroups. Ten genes in *Arabidopsis* and 38 genes in *B. napus* were included in the *NF-YA* subfamily, 13 genes in *Arabidopsis* and 46 genes in *B. napus* were included in the *NF-YB* subfamily, and 13 genes in *Arabidopsis* and 24 genes in *B. napus* were included in the *NF-YC* subfamily. The *NF-Y* genes in the same subfamily were closely related to each other. Furthermore, some genes of the *NF-Y* group in *Arabidopsis* did not have homologous genes in *B. napus*, while some other genes of the *Arabidopsis NF-Y* groups had two or more homologous genes in *B. napus*. This may be due to the loss of certain genes and the homologous replication of chromosomes during evolution. To distinguish each subfamily of the *NF-Y* genes, all the *BnaNF-Y* members were renamed according to the *Arabidopsis* homologous genes following the international nomenclature from *BnaA02.NF-YA1* to *BnaC07.NF-YC13* (Figure S1, Table S3).

### 2.4. Structural Analysis and Conserved Motif Analysis of the NF-Y Genes in B. napus and Arabidopsis

**Figure 4:**
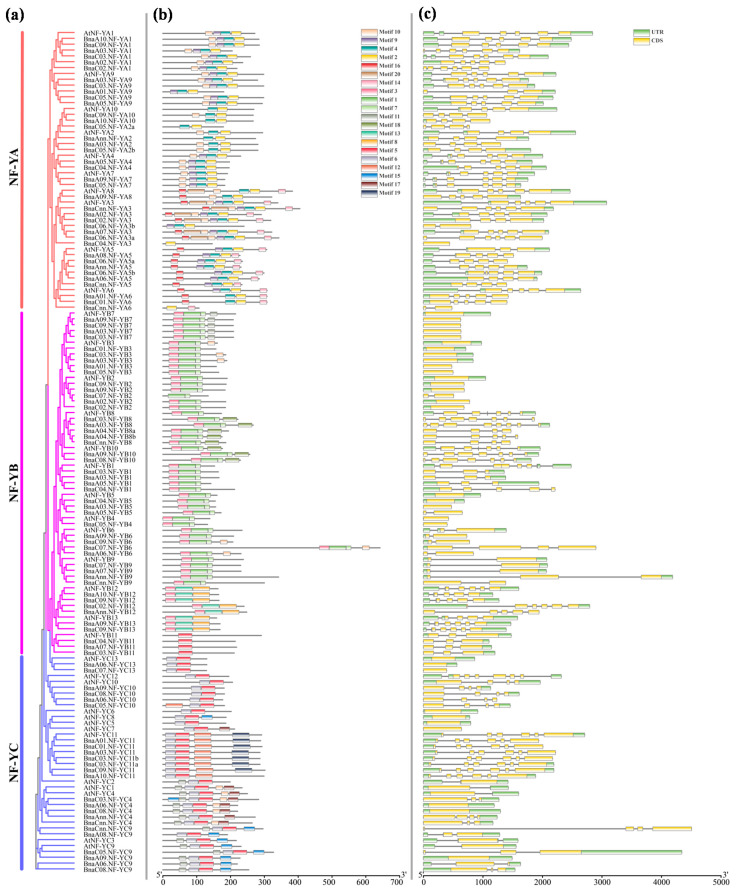
Phylogenetic relationships, gene structure and conserved motif compositions of the *NF-Y* family in *B. napus* and *Arabidopsis*. (**a**) The phylogenetic relationships of the *BnaNF-Y* and *AtNF-Y* genes. The 108 genes of *B. napus* and 36 genes of *Arabidopsis* are divided into three groups; (**b**) Conserved motif compositions. Each colored box in the middle represents a motif, and the black line represents non-conserved sequences; (**c**) Gene structure. The untranslated region (UTR), exon and intron in the right of each subfamily are represented by a green box, yellow box and gray line, respectively.

The gene structure of the 144 *NF-Y* family members in *B. napus* and *Arabidopsis* were further analyzed. The results showed that the gene structure varied largely among different subfamilies, while it was relatively conserved in the same family (Figure 4, Table S3). The number of introns varied from 0 to 7 with an average number of 2.6. Twenty-two *NF-Y* genes had no intron, while *BnaA03.NF-YB8* had eight introns, the largest number among all the members. Generally, the *BnaNF-Y* genes had similar numbers of introns as their counterparts in *Arabidopsis*. In addition, we analyzed the motif structures of the *NF-Y* family genes using their amino acid sequences in MEME. A total of 20 conserved motifs were identified, and the number of amino acids in each motif ranged from 6 to 50 (Figure 4 and Figure S2). Similar to gene structure, nearly all the *NF-Y* genes in the same subfamily had relatively conserved motif composition and arrangement; however, it varied to a substantial degree in different subfamilies, especially among the *NF-YA*, *NF-YB* and *NF-YC* subfamilies. In addition, each subfamily contains specific motifs. In detail, the *NF-YA* subfamily specifically contains motifs 2, 4, 9, 14, 16 and 20, *NF-YB* subfamily specifically contains motif 1, 3, 7, 8, 13 and 18, and *NF-YC* subfamily specifically contains motif 6, 12, 15, 17 and 19, and they all have motif 10 (Figure 4 and Figure S2). Taken together, the results of the structural analysis and conserved motif analysis strongly demonstrate the reliability of the genetic identification results.

### 2.5. Chromosomal Location and Gene Duplication Patterns of the NF-Y Genes in B. napus

Chromosomal location analysis revealed that the 108 *BnaNF-Y* genes were unevenly distributed across ten chromosomes in the A subgenome (51 genes) and nine chromosomes in the C subgenome (57 genes) of *B. napus* (Figure S3). Among them, the specific location of *BnaA10.NF-YA1* on chromosome A10 was unknown, while five genes (*BnaAnn.NF-YA2*, *BnaAnn.NF-YB9*, *BnaAnn.NF-YB12*, *BnaAnn.NF-YC4* and *BnaAnn.NF-YA5*) and seven genes (*BnaCnn.NF-YC9*, *BnaCnn.NF-YC4*, *BnaCnn.NF-YA5*, *BnaCnn.NF-YA6*, *BnaCnn.NF-YB9*, *BnaCnn.NF-YA3* and *BnaCnn.NF-YB8*) could not be mapped to a specific chromosome of *B. napus*. Chromosome C03 contained the largest number of the *NF-Y* family members, followed by A03, A09, C05 and C09. Chromosomes A08 and A04 had the smallest number of the *NF-Y* genes.

**Figure 5:**
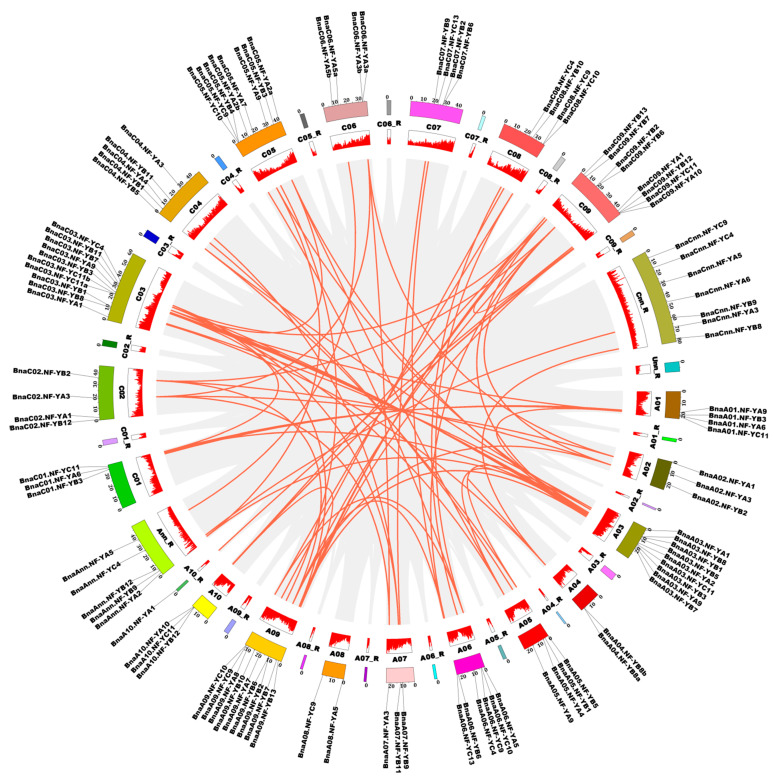
Schematic representations for the chromosomal distribution and interchromosomal relationships of the rapeseed *NF-Y* genes. The chromosomal localizations are shown in the outer cycle. The sequence length of each *B. napus* chromosome is indicated by the numbers along the box. The *BnaNF-Y* genes are mapped on the different chromosomes. Gene density of the whole *B. napus* genome is shown in the inner cycle. The higher the red column in the inner circle, the more genes there are on the chromosome. Gray lines inside the cycle indicate the genome-wide collinearity within the *B. napus* genome, while the red lines show the syntenic *BnaNF-Y* gene pairs. R: random.

To unravel the evolutionary processes of the 108 *NF-Y* genes in oilseed rape, the gene expansion patterns including tandem and segmental duplication were further analyzed using the coding sequences. A total of 144 segmental duplication events were identified using MCScanX methods (Figure 5). The *NF-Y* genes in the A subgenome of *B. napus* had high homology with the *NF-Y* genes in the C subgenome. For example, two genes (*BnaA03.NF-YB7* and *BnaA03.NF-YC11*) located on chromosome A03 had strong homology with two genes (*BnaC03.NF-YB7* and *BnaC03.NF-YC11a*) located on the chromosome C03. Two genes (*BnaA09.NF-YB2* and *BnaA09.NF-YB6*) located on chromosome A09 are highly homologous to two genes (*BnaC09.NF-YB2* and *BnaC09.NF-YB6*) on chromosome C09. However, only two tandem duplication events were observed. These results indicate that the major driving force for the expansion of the *NF-Y* family genes in *B. napus* might be segmental duplication events.

**Figure 6:**
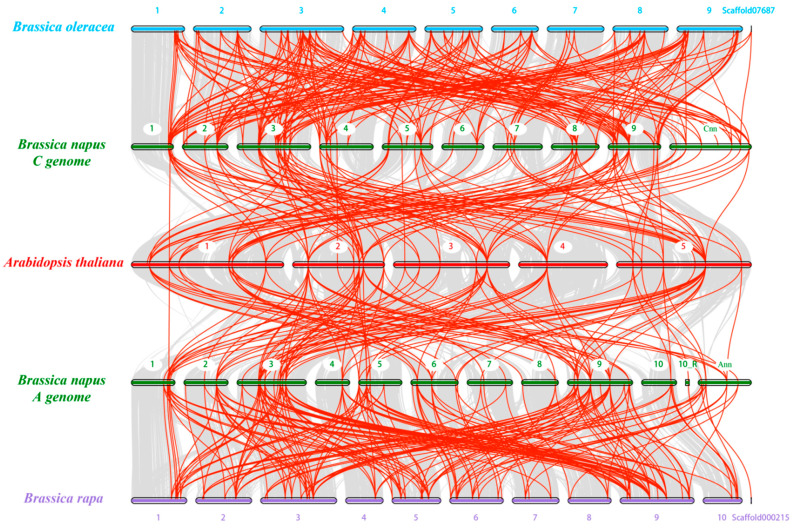
Collinearity analysis of the *NF-Y* family genes in *B. napus*, *B. rapa*, *B. oleracea* and *Arabidopsis* chromosomes. The solid bars with different colors represent different chromosomes of the four species. The gray lines in the background suggest the syntenic blocks between the genomes of two species for comparison, while the red lines indicate the syntenic *NF-Y* gene pairs. R: random.

Furthermore, a comparative syntenic map between the *NF-Y* genes in *B. napus* and *Arabidopsis* was constructed to further deduce the expansion mechanisms of the *B. napus NF-Y* family members. The results showed that there were strong orthologs among *B. napus*, *B. rapa*, *B. oleracea* and *A. thaliana* (Figure 6). In detail, 110 and 187 pairs of collinear relationships were observed in the A subgenome of *B. napus* with *A. thaliana* and *B. rapa*, respectively. Additionally, the C subgenome of *B. napus* contained 114 and 219 pairs of syntenic relationships with *A. thaliana* and *B. oleracea*, respectively. In order to better understand the evolutionary constraints acting on the *NF-Y* genes between *B. napus* and *A. thaliana*, the nonsynonymous (Ka)/synonymous (Ks) ratios were estimated. The results showed that the Ka/Ks ratios of all the *NF-Y* gene pairs between *B. napus* and *A. thaliana* were less than one (Figure 3d–f, Table S4), suggesting that the *NF-Y* genes in rapeseed might have suffered robust purifying selective pressure during evolution.

### 2.6. Expression Patterns of the BnaNF-Y Genes in Response to Different Nutrient Deficiencies

**Figure 7:**
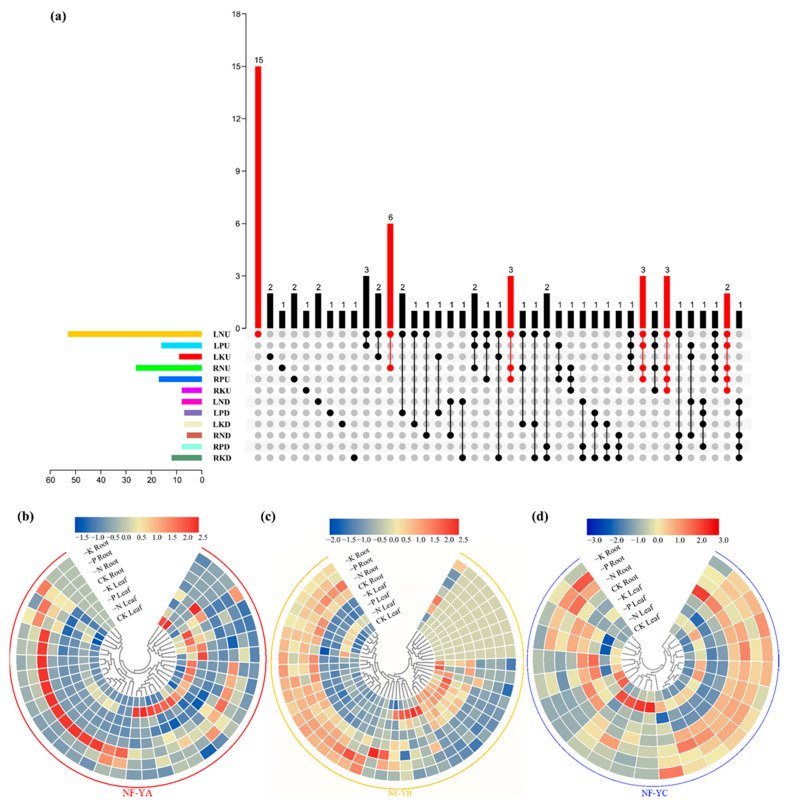
Expression profiles of each *NF-Y* subfamily in rapeseed leaf and root under three nutrient deficient conditions. Seedlings 14 days old were cultivated under nitrogen, phosphorus and potassium deficient environments, respectively, for 6 days. The fully expanded leaf and root were harvested separately for RNA extraction. (**a**) Upset plot of significantly regulated genes under different treatments in two tissues. The red column represents the treatment with the highest number of genes in the same number of crosses; (**b**) The *BnaNF-YA* subfamily; (**c**) The *BnaNF-YB* subfamily; (**d**) The *BnaNF-YC* subfamily. The color scale is shown above each subfamily. The data in the heat map was normalized (z-scores) using FPKM values and then generated by Tbtools; the higher the value of the color scale, the higher the expression, and vice versa. −N: nitrogen deficiency; −P: phosphorus deficiency; −K: potassium deficiency; CK: sufficient nutrient supply. L: leaf; R: root; N: nitrogen deficiency; P: phosphorus deficiency; K: potassium deficiency. D: downregulated; U: upregulated.

To check the functions of the *BnaNF-Y* genes in response to various nutrient limitations, the expression profiles of the 108 *BnaNF-Y* members were analyzed under N, P or K deficiencies as well as sufficient nutrient supply conditions using RNA-seq data. The results demonstrated that the expression levels of the *BnaNF-Y* genes changed largely in response to different nutrient deficiencies and varied among different subfamilies (Table S5, Figure 7 and Figure S4a). In leaf, the expression of 61, 23 and 16 genes were significantly changed under N, P and K starvations, respectively (Figure S3b). In root, 32, 25 and 20 genes were affected dramatically by N, P and K deficiencies, respectively (Figure S3c). Some of the differentially expressed genes (DEGs) responded specifically to a certain nutrient deprivation, while others changed in response to two or three nutrient deprivations (Figure S3b,c). Among all the DEGs, 86.89% (53/61), 69.57% (16/23) and 56.25% (9/16) of the *BnaNF-Y* genes were upregulated by N, P and K deficiencies in leaf, respectively (Figure S3d). In root, 81.25% (26/32), 68.00% (17/25) and 40.00% (8/20) of the DEGs were enhanced by N, P and K deficiencies, respectively (Figure S3e). The rest of the DEGs in leaf and root were downregulated by different nutrient limitations (Figure S3d,e). Further analysis of the DEGs showed that 34 genes were regulated only in one nutrient deficiency condition in both leaf and root, including 26 under N deficiency, three under P deficiency, and five under K deficiency (Figure 7a and Table S5). A total of 26 DEGs responded specifically to a certain environment in one organ (Figure 7a). Fifteen DEGs were specifically upregulated by N deficiency in leaf, and six DEGs were specifically induced by N starvation in both leaf and root (Figure 7a), indicating their key roles in N deficiency response. Twelve *BnaNF-Y* genes were upregulated in one nutrient deficiency treatment but downregulated in another (Figure S6), indicating their opposite roles in regulating different nutrient starvation responses.

**Figure 8:**
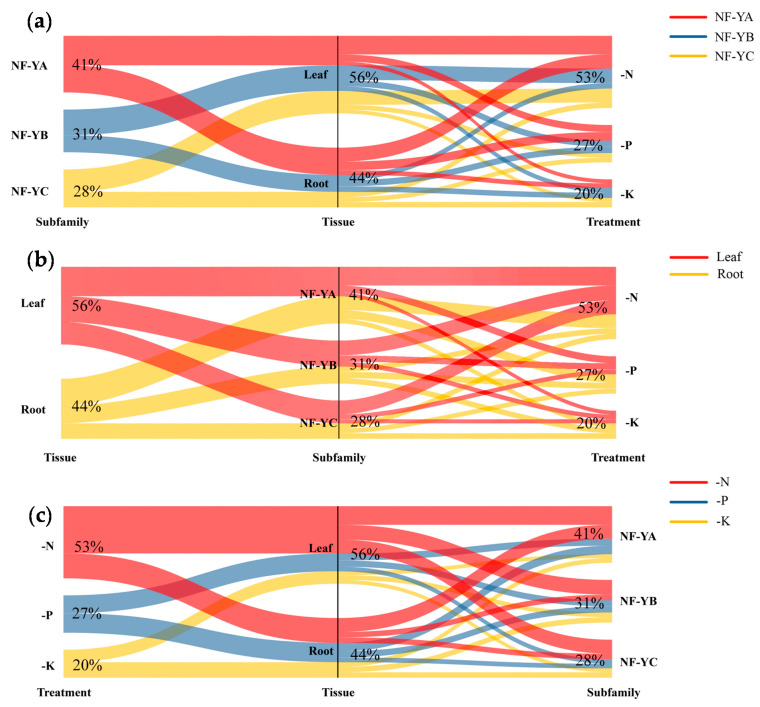
Genome distribution map of the differentially expressed *NF-Y* genes in two tissues under three nutrient starvations. Sankey diagram is drawn to depict the ratios of the differentially expressed *NF-Y* genes in one triad. (**a**) Three *NF-YA* subfamilies; (**b**) Two tissues; (**c**) Three nutrient deficiencies. -N: nitrogen deficiency; -P: phosphorus deficiency; -K: potassium deficiency. Distinct colors represent the flow of triads belonging to one category into the other two categories. The ratios of each triad are shown on the map.

Since the *NF-Y* family is a heterotrimeric complex with three distinct subfamilies, we further analyzed the expression pattern of each subfamily in response to N, P and K limitations. The results showed that 68.42% (26/38), 36.96% (17/46) and 33.33% (8/24) of the *NF-YA*, *NF-YB* and *NF-YC* genes, respectively, were differentially expressed under nutrient limitation conditions (Figure 7b–d). To further characterize the response of the three *BnaNF-Y* subfamilies to N, P and K starvations in two organs, we calculated the ratios of DEGs under different contexts and visualized the data using Sankey diagrams (Figure 8). In terms of the three *NF-Y* subfamilies, the differentially expressed *NF-YA*, *NF-YB* and *NF-YC* genes accounted for 41% (73/177), 31% (55/177) and 28% (49/177), respectively (Figure 8a). For the two organs, 44% (77/177) DEGs were detected in root, and 56% (100/177) in leaf (Figure 8b). Moreover, 53% (93/177), 27% (48/177) and 20% (36/177) of the total DEGs responded to N, P and K deficiencies, respectively (Figure 8c). These results suggest that the *NF-YA* subfamily may play a more important role in response to nutrient deprivation as compared to the *NF-YB* and *NF-YC* subfamilies, especially in N deficient environments.

### 2.7. Discovery of the NF-Y Hub Genes in B. napus and Expression Analysis of these Genes among Different Tissues under N Limitation

**Figure 9:**
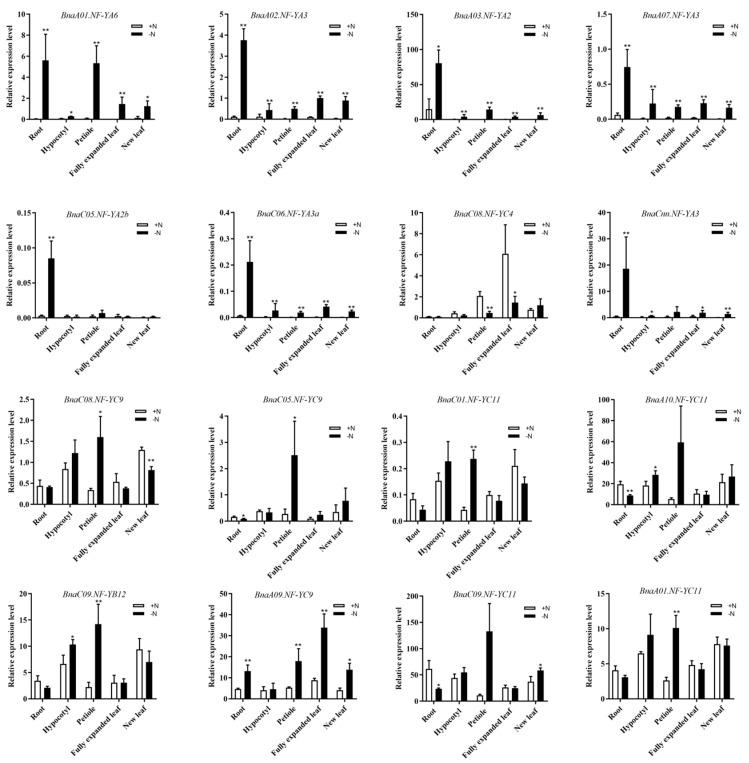
The expression levels of the 16 selected *BnaNF-Y* genes in five tissues of *B. napus* in response to nitrogen (N) deficiency by qRT-PCR. Fourteen-day-old rapeseed seedlings were cultivated in a nutrient solution free of N for six days. RNA was extracted separately from five tissues including root, hypocotyl, petiole, fully expanded leaf and new leaf under N sufficient and deficient conditions. +N: N sufficient condition; −N: N free (0 μM N) condition. Each sample included three independent biological replicates. * and ** indicate significant differences at *p* < 0.05 and *p* < 0.01 per student’s *t* test, respectively.

To reveal the coexpression relationships among the *BnaNF-Y* genes, we calculated the interaction weight of the target genes based on the fragments per kilobase million (FPKM) values of the RNA-seq data under N, P and K starvation conditions (Figure S7). Generally, we identified 1091 pairs of coexpression relationships among the 108 *BnaNF-Y* family genes. Then, the *BnaNF-Y* genes with the ten strongest interaction relationships with other *BnaNF-Y* members were considered as the hub genes, as displayed in Figure S7. As shown in Figure 8, more than half (53%) of the DEGs were detected under N deficiency. Hence, to understand the detailed expression profiles of the *BnaNF-Y* family genes in response to N limitation, we further investigated the expression levels of the 16 core genes in root, hypocotyl, basal node, petiole, fully expanded leaf and new leaf of *B. napus* using quantitative real-time PCR (qRT-PCR). The results illustrated that the mRNA levels of these genes in oilseed rape were basically consistent with the results from the RNA-Seq data (Figure 9, Table S5). Some genes, such as *BnaC05.NF-YA2b*, *BnaCnn.NF-YA3* and *BnaC06.NF-YA3a*, were specifically induced by N starvation in root and some had high expression levels in fully expanded leaf, such as *BnaC08.NF-YC4* and *BnaA09.NF-YC9*. Additionally, the core *NF-Y* genes were expressed differently in tissues other than root and leaf in response to N deficiency (Figure 9). For example, the expression levels of *BnaA01.NF-YA6*, *BnaC08.NF-YC9*, *BnaC05.NF-YC9*, *BnaC09.NF-YB12*, *BnaA01.NF-YC11* and *BnaC01.NF-YC11* were significantly induced in petiole by N deficiency.

### 2.8. Subcellular Localization of Four NF-Y Hub Genes in Rapeseed

**Figure 10:**
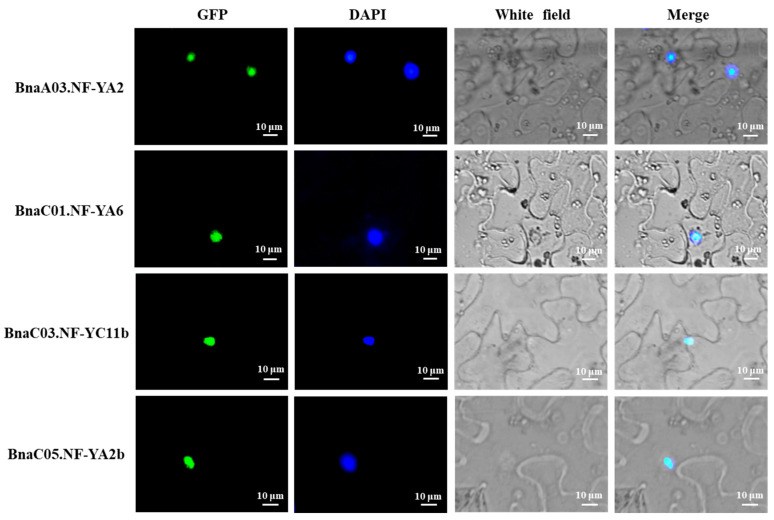
Subcellular localization of *BnaA03.NF-YA2*, *BnaC01.NF-YA6*, *BnaC03.NF-YC11b* and *BnaC05.NF-YA2b.* 35S::BnaNF-Ys-GFP constructs were introduced into tobacco. The blue color shows nuclei by DAPI staining. The GFP and DAPI fluorescence was observed with a fluorescence microscope. The images were taken in the dark and white field.

We further selected four *NF-Y* hub genes in rapeseed (*BnaA03.NF-YA2*, *BnaC01.NF-YA6*, *BnaC03.NF-YC11b* and *BnaC05.NF-YA2b*) to explore their subcellular localization. The full length cDNA of four *BnaNF-Y* genes was isolated and integrated with PMDC83 vector to form 35S::BnaNF-Ys-GFP protein structure. Then, these constructed vectors were injected into tobacco leaves. Meanwhile, we used nuclear dye 4′,6-diamidino-2-phenylindole (DAPI) for auxiliary labeling of nuclei. The results suggested that all of the GFP fusion protein signals were completely merged with the DAPI signals (Figure 10). Consistent with their characteristics as transcription factors, these *BnaNF-Y* genes were located in the nucleus. Combined with our above analysis, these results jointly emphasized the important roles of *NF-Y* genes in regulating plant responses to nutrient deficiencies, especially N deprivation.

## 3. Discussion

The *NF-Y* genes are trimeric transcription factors in plants, containing the *NF-YA*, *NF-YB* and *NF-YC* subfamilies which include multiple genes [2]. Recently, the *NF-Y* families have been identified and characterized in several plant species, including *Arabidopsis* [5], rice [6], soybean [7], sorghum [10], barley [13], apple [15] and more. Previously, the *NF-Y* genes were identified and characterized in *B. napus* based on an expressed sequence tag database and the completed *B. napus* genome sequence in the CoGe database [42,43]. However, there had been no comprehensive genome-wide investigation or systematic characterization in *Brassica* species. In the present study, 62, 54 and 108 *NF-Y* genes were identified in *B. rapa*, *B. oleracea* and *B. napus*, respectively (Table S1). Especially in *B. napus*, the number of *NF-Y* family members is far greater than that found in other species (Table S2) or in the two previous reports on *B. napus* [42,43]. Therefore, substantial changes via evolution and selection must have occurred during the evolution processes of *Brassica* species. Moreover, we found that such purifying selective pressure may have been a strong driving force in the evolution of *Brassica* species (Figure 3e, Table S4). Interestingly, and similar to other species, the Ka/Ks ratios of all the *NF-Y* gene pairs were less than one in *B. napus*, suggesting that the *NF-Y* family genes may be conserved in function [10]. The gene replication pattern analysis showed that 144 segmental duplication events occurred in the *B. napus* genome; however, only two tandem duplication pairs were observed (Figure 5). In rice, the *NF-Y* family expansion was mainly driven by segmental duplication events, which indicates that the *NF-Y* family might have similar expansion patterns in dicotyledons and monocots [6,32].

Moreover, the conserved domain analysis of the *NF-Y* subfamilies in three *Brassica* species and *Arabidopsis* indicates that the *NF-YC* subfamily excepted, the *NF-YA* and *NF-YB* subfamilies are relatively conservative (Figure 2). Both the *NF-YA* and *NF-YC* subfamilies in *B. oleracea* showed large differentiation compared with others, which further provides strong evidence for an obvious selection during the evolution of *Brassica* species. Similarly, the gene structure and motifs differed across different subfamilies in *B. napus*, which is consistent with previously reports in sorghum [10], citrus [12] and tea [44]. However, the *NF-Y* genes in the same subfamily, such as *NF-YB11s*, had a similar composition and arrangement of structure and motifs, suggesting that the function of genes in each subfamily may be relatively conserved in *B. napus* (Figure 4, Table S3). Generally, these results put forward new insights into the phylogenetic mechanism of the *NF-Y* family in *Brassica* species.

Over the past decade, the *NF-Y* genes as the key regulators of drought resistance in plants have been widely investigated [34]. Previous studies showed that over-expression of the *NF-Y* genes could enhance drought tolerance in *Arabidopsis* [45,46] and *Populus* [47]. However, fewer studies focused on the relationships between the *NF-Y* genes and plant stress resistance under nutrient limitation conditions. Here, we investigated the expression of the *NF-Y* family in *B. napus* in response to multiple nutrient limitations. The results suggest that the mRNA levels of the *BnaNF-Y* family members display various expression patterns under distinct nutrient deficiencies, which may be related to their complex structure and motif compositions (Figure 7 and Figure S4a, Table S5). In soybean, *NF-YA12* was upregulated by abscisic acid, NaCl and cold stresses [7]. Likewise, several *NF-Y* genes were induced under multiple stress conditions in sorghum [11]. Interestingly, except for the *BnaNF-Y* genes (whose expression levels were regulated by different nutrient deficiencies) we also found that the expression changes of 34 genes were stress-specific (Figure 7 and Figure S5). Among them, the expression levels of seventeen, one and three *BnaNF-Y* genes were specifically regulated in leaf under N, P and K deprivations, respectively, while only five genes which specifically responded to a certain nutrient deficiency were observed in roots (Figure 7). Thus, that these *NF-Y* genes responded specifically to a certain tissue under a specific nutrient deficiency may indicate a unique role in improving stress resistance in *B. napus*. In addition, reports show that ABA-dependent or independent signaling pathways are involved in the response of many *NF-Y* genes to abiotic stresses [31,48]. In the current research, the expression levels of 12 *BnaNF-Y* genes were inversely regulated under multiple nutrient deprivations, indicating that they might participate in completely different regulatory pathways in order to regulate plant growth. However, whether the regulatory pathways associated with the *BnaNF-Y* genes are ABA-dependent or independent still needs to be further verified.

In sorghum, the expression profiles of the three *NF-Y* subfamily members differed largely in response to various abiotic stresses, suggesting that different *NF-Y* subfamily members might have specific subfunctionalization [11]. Similar results were observed here, in that the *BnaNF-YA* family genes had the most obvious response to multiple nutrient starvations (Figure 7b–d and Figure 8). Consistent with previous studies, the *NF-YA* family members were the genes most related to regulation of plant nutrient deficiency responses. For example, three *Arabidopsis NF-YA* genes (*AtNF-YA2*, *AtNF-YA7* and *AtNF-YA10*) might be post-transcriptionally regulated by *miR169* to regulate plant tolerance to P deficiency [35]. In addition, the absorption of N and P as well as grain yield were significantly enhanced in the *TaNFYA-B1* over-expression lines under N and P deficient levels in wheat [37]. Therefore, compared with other subfamily members the *NF-YA* subfamily might act as a more critical player in response to nutrient deficiencies in *B. napus*. Among these, the responses of the *BnaNF-YA* family to nutritional starvations were slightly changed compare to that in *Arabidopsis*. For instance, the expressions of *AtNF-YA2*, *AtNF-YA3* and *AtNF-YA5* were be induced by P deficiency [49], while their homologous genes in *B. napus* were nearly unaffected by P limitation (Table S5). However, the three *NF-YA* genes were upregulated by N deprivation in both *Arabidopsis* and *B. napus* [36], indicating that they still retained some important functions in *B. napus* (Table S5). N deficiency significantly inhibited the growth of *B. napus* compared with other nutrient deficiencies, and strongly regulated the expression of most *NF-Y* genes (Figure 1 and Figure 8). Thus, the results fully suggest that the *BnaNF-Y* family genes may have important roles in response to N limitation in oilseed rape, especially the *NF-YA* subfamily (Figure 8).

The homologous genes of other species contained more than one copy compared with *Arabidopsis*, and might have experienced functional differentiation. In *B. napus*, the complex evolutionary processes of the *NF-Y* family genes give rise to its functional diversification under multiple nutrient deficiencies. To investigate the expression changes of the *BnaNF-Y* genes in response to different nutrient limitations in more details, we identified 10 hub genes based on the coexpression network analysis (Figure S7). Besides the *NF-YA* and *NF-YC* subfamilies that have been reported to be closely related to nutrient deficiencies, another four *BnaNF-YB* genes also displayed strong relationships (Figure S7). Reports showed that *AtNF-YA2, AtNF-YA3, AtNF-YA5* and *AtNF-YA8* were post-transcriptionally regulated by *miR169a* to enhance plant adaptability to low N availability in soil, while *AtNF-YC4* was related to N and carbon distribution in plants [36,49]. Furthermore, the sensitivity of the *NF-Y* family genes to N starvation was higher than that to P and K starvations in *B. napus* (Figure 8). Thus, we further investigated the mRNA levels of the *BnaNF-Y* hub genes and other *BnaNF-Y* genes specifically responding to N deficiency in different tissues. In line with other species, most *NF-Y* genes revealed intense tissue specificity, and seven *BnaNF-YA* subfamily genes were mainly induced by N deficiency in root (Figure 9). Such tissue-specific expression patterns have been observed in a few plants [11,50]. Interestingly, *BnaA10.NF-YC11* was significantly induced in hypocotyl by N deficiency, but was inhibited in root (Figure 9), suggesting that this gene might play different roles in specific tissues under N deficiency. In addition, unlike other genes, *BnaC08.NF-YC4* was significantly inhibited in petiole and fully expanded leaf (Figure 9). Meanwhile, *BnaNF-Ys* as a transcription factor was located in the nucleus (Figure 10). This evidence indicates that the *BnaNF-Y* genes might participate in modulating nutritional stress tolerance through positive or negative mechanisms, which have been testified to in *Arabidopsis* [35].

## 4. Materials and Methods

### 4.1. Identification of the NF-Y Family Genes in B. napus

The *NF-Y* family members of *B. napus* were obtained using BLASTP in the CNS-Genoscope genomic database (http://www.genoscope.cns.fr/brassicanapus/, accessed on 1 September 2019) based on the protein sequences of 36 *Arabidopsis NF-Y* genes from TAIR database (https://www.arabidopsis.org/, accessed on 1 September 2019). The homologous genes in three *Brassica* species were obtained based on E-value < 1 × 10^−5^. The redundant gene sequences of the identified *Brassica* species were removed manually. In order to ensure the reliability of identified genes, the Hidden Markov Model (HMM) in the Pfam database (http://pfam.xfam.org/, accessed on 1 September 2019) was employed with E-value < 0.001 to eliminate out-of-standard genes, which also did not contain the complete CBFD-NFYs domain (PF02045 and PF00808). The full genomic sequences, coding sequences and protein sequences of *B. napus NF-Y* members were derived from the *B. napus* genome database. For *B. rapa* and *B. oleracea*, the same methods described above were used to obtain the sequence information of *NF-Y* family members from the *Brassica* Database (BRAD, http://brassicadb.org/brad, accessed on 1 September 2019).

### 4.2. Chromosomal Location and Gene Duplication Analysis of the NF-Y Genes in B. napus

The chromosomal locations of the *NF-Y* genes were determined from the *B. napus* genome database and were displayed by TBtools [51]. Gene duplication events and collinearity relationships of the *NF-Y* genes in *Arabidopsis* and three *Brassica* species were analyzed using Multiple Collinearity Scan toolkit (MCScanX) [52]. The standard for identifying potential gene duplication was defined by the following two points: (a) the comparison region covers more than 75% of the gene; and (b) the similarity of the aligned regions is more than 75%. Likewise, the gene duplication events and collinearity relationships of the *NF-Y* genes between *B. oleracea* and *B. napus* or between *B. rapa* and *B. napus* were analyzed using the same methods described above. MCScanX was employed to construct the map which was used to demonstrate the syntenic relationships between the *B. napus NF-Y* family genes and its ancestors in *Arabidopsis*, *B. rapa* and *B. oleracea*.

### 4.3. Phylogenetic Relationships and Evolutionary Pressure Analysis of the B. napus NF-Y Genes

MEGA7 software was employed to conduct the protein sequence alignments with default parameters, and the evolutionary tree analysis tool was used to build phylogenetic trees with the neighbor joining (NJ) method based on 1000 bootstraps [53]. In order to analyze the selection pressure of *BnaNF-Y* family evolution, values of the Ka, Ks and Ka/Ks ratio between *Arabidopsis* and *B. napus* were calculated based on the sequence alignments. Pairwise alignments of the gene CDS without stop codon were performed to calculate Ka/Ks ratio by the Ka/Ks calculator in TBtools software [51]. Generally, a Ka/Ks ratio greater than one indicates positive selection, while a Ka/Ks ratio less than one suggests a functional constraint; equal to one indicates neutral selection [54].

### 4.4. Molecular Characterization Analysis of the B. napus NF-Y Family Genes

The full genomic sequences, coding sequences and protein sequences of the *BnaNF-Y* genes downloaded from the CNS-Genoscope genomic database were used to analyze gene structure by multiple alignments. The gene structure was then visualized in TBtools [51]. The potential conserved motifs of the *NF-Y* family genes in *Arabidopsis* and *B. napus* were detected using Multiple Expectation Maximization for Motif Elicitation program (MEME, http://meme-suite.org/tools/meme, accessed on 3 June 2019) with the following four parameters: (a) the maximum input data was 6000; (b) the analysis mode was anr; (c) the optimum motif width ranged from 6 to 50; and (d) the maximum motif number was 20 [55]. Moreover, the MW and pI of *BnaNF-Y* proteins were calculated using ExPASy tool (http://www.expasy.org/tools/, accessed on 3 June 2019), and the GRAVY values of the proteins were analyzed using the PROTPARAM tool (http://web.expasy.org/protparam, accessed on 3 June 2019). WoLF PSORT server (https://wolfpsort.hgc.jp/, accessed on 3 June 2019) was employed to predict the subcellular localizations of the *BnaNF-Y* proteins.

### 4.5. Heat Map and Coexpression Networks of the BnaNF-Y Family Genes Using RNA-seq Data

The heat map and gene coexpression network analysis were performed based on the RNA-seq data. In the RNA-seq experiment, 14-day-old rapeseed seedlings were treated with N-free, P-free and K-free nutrient solution for 10 days. Then, the fully expanded leaf and root were harvested separately for RNA extraction. After harvest, all samples were frozen immediately in liquid N and then stored at −80 °C. Each treatment included three independent biological replicates. Illumina HiSeq 2500 platform (Illumina, San Diego, CA, USA) was applied to perform transcriptome sequencing. The FPKM values indicating the transcript abundance of all genes under control and N/P/K deficient conditions were obtained based on gene length and the reads mapped to the gene. Moreover, DEGs were identified based on *p* value < 0.05 between control and treatments. The interactions and weight values of the target genes were analyzed using DeGNServer online tools (http://plantgrn.noble.org/DeGNServer/Analysis.jsp, accessed on 9 October 2019) based on the FPKM values. Cytoscape software was employed to visualize the coexpression networks of the *BnaNF-Y* genes [56].

### 4.6. Plant Materials and Growth Conditions

A *B. napus* cultivar named “ZS11” was planted in an illuminated growth room (22 °C; 16 h light/8 h dark) with a light intensity 300–320 mmol protons m^−2^ s^−1^. The seeds were first disinfected and soaked in deionized water in the dark for two days, then transferred to 0.5 mM CaCl_2_ solution for three days to promote root development. Finally, modified Hoagland’s solution with pH 5.8 was used for seedling growth, containing 5.0 mM Ca(NO_3_)_2_, 5.0 mM KNO_3_, 1.0 mM KH_2_PO_4_, 2.0 mM MgSO_4_, 46.0 μM H_3_BO_3_, 9.0 μM MnCl_2_, 0.3 μM CuCl_2_, 0.8 μM ZnCl_2_, 0.32 μM Na_2_MoO_4_ and 50.0 μM EDTA-iron (Fe). After nine days’ growth, plants were treated with different nutrient concentrations for six days, including N, P and K deficiencies. CaSO_4_ and K_2_SO_4_ were used to substitute Ca(NO_3_)_2_ and KNO_3_ in N starvation treatment, respectively. K_2_SO_4_ was replaced by KH_2_PO_4_ in P starvation treatment. In K starvation treatment, NaH_2_PO_4_ and NaNO_3_ were used to replace KH_2_PO_4_ and KNO_3_, respectively. Tissue samples were collected after the treatments and stored at −80 °C until RNA extraction. Three biological replicates were prepared for each treatment. For phenotypic analysis, the *B.* *napus* cultivar “ZS11” was cultivated for 11 days under low N (LN, 100 μM), low P (LP, 5 μM) and low K (LK, 5 μM) conditions (Figure 1). Then, the taproot root length, shoot and root dry weight were measured. SPAD value was measured by SPAD meter (SPAD 502 plus, Minolta, Japan). Photosynthetic parameters were detected by Photosynthesis System (CIRAS-3, America).

### 4.7. Expression Analysis of the BnaNF-Y Genes under Multiple Nutrient Deficiencies

A RNeasy Plant Mini Kit (Qiagen) was used to extract the total RNA of each sample, and a First Strand cDNA synthesis kit (Toyobo) was employed to synthesize cDNA. The qRT-PCR was carried out in a 10 μL volume containing 2.5 μL cDNA, 0.2 μL primers, 5 μL Hieff qPCR SYBR Green Master Mix (Low Rox). The thermal cycle was as follow: 95 °C 5 min; 40 cycles of 95 °C for 10 s, 60 °C for 20 s, 72 °C for 20 s. qRT-PCR was performed using the QuantStudio 6 Flex instrument (Life Technologies, USA). The relative gene expression levels were calculated according to the 2^−ΔΔ*Ct*^ method [57]. The housekeeping gene *EF1-α* (Accession number DQ312264) was used as an internal standard [58]. The primers used for qRT-PCR in this study are listed in Table S6.

### 4.8. Subcellular Localization Analysis of the BnaNF-Y Genes

For subcellular localization, the coding regions of four *BnaNF-Y* genes without stop codons amplified from “ZS11” were ligated separately into the vector (PMDC83) driven by the CaMV35S promoter. DAPI was used to stain nuclei; they were cultured on LB agar supplemented with selection antibiotics and incubated at 28 °C for two days. The confluent *Agrobacterium* with the target vector were resuspended in infiltration buffer (10 mM MgCl_2_, 0.5M MES (pH 5.6), 100 mM acetosyringone) to an optical density (600 nm) of 1.0, and incubated at room temperature without shaking for 3 h before infiltration. Approximately 2 mL of the *Agrobacterium* mixture was then infiltrated into the back of 3-4 young leaves of tobacco. The subcellular localization assay was performed 2 d after inoculation [59]. A fluorescence microscope was used to record the images.

### 4.9. Statistical Analysis

Student’s *t* test was used to perform the statistics in SPSS 22 (IBM, Chicago, IL, USA). Significance of differences was defined as * *p* < 0.05, ** *p* < 0.01.

## Data Availability

The data presented in this study are available on request from the corresponding authors.

## Supplementary Materials

The following are available online at https://www.mdpi.com/article/10.3390/ijms221910354/s1, Figure S1: Phylogenetic tree of the *NF-Y* family in *B. napus* and *Arabidopsis*. The analysis includes 144 protein sequences, including 108 from *B. napus* (white star) and 36 from *Arabidopsis* (light green circle). The different colors mean different subfamilies. Figure S2: The sequence information of the *NF-Y* family protein for each motif in *Arabidopsis* and *B. napus*. Figure S3: Chromosomal distribution of the *NF-Y* family members in *B. napus*. The localization of each *BnaNF-Y* gene on the chromosome was represented via colored fonts. Bar = 5 Mb. Figure S4: The expression map of all *B. napus NF-Y* genes: (a) The heat map of all *B. napus NF-Y* genes. The color scale is shown above the expression map. The data in the heat map was normalized (z-scores) using FPKM values and then visualized by Tbtools. The higher the value of the color scale, the higher the expression, and vice versa; (b) Venn diagram of the differentially expressed genes (DEGs) among three nutrient deficiencies in leaf; (c) Venn diagram of the DEGs among three nutrient deprivations in root; (d) The number of total, up- and downregulated DEGs in response to three nutrient deficiencies in leaf; (e) The number of total, up- and downregulated DEGs in response to three nutrient limitations in root. CK: sufficient nutrient supply; -N: nitrogen deficiency condition; -P: phosphorus deficiency condition; -K: potassium deficiency condition. Figure S5: Heat map of the *B. napus NF-Y* family genes differentially expressed in only one treatment based on the RNA-seq data. The color scale is shown above each subfamily. The data in the heat map was normalized (z-scores) using FPKM values and then visualized by TBtools. The higher the value of the color scale, the higher the expression, and vice versa. CK: sufficient nutrient supply; -N: nitrogen deficiency condition; -P: phosphorus deficiency condition; -K: potassium deficiency condition. * and ** indicate significant differences at *p* < 0.05 and *p* < 0.01 per student’s *t* test, respectively. Figure S6: Heat map of the *B. napus NF-Y* family genes differentially expressed in two or three treatments based on the RNA-seq data. The color scale is shown above each subfamily. The data in the heat map was normalized (z-scores) using FPKM values and then visualized by Tbtools. The higher the value of the color scale, the higher the expression, and vice versa. CK: sufficient nutrient supply; -N: nitrogen deficiency condition; -P: phosphorus deficiency condition; -K: potassium deficiency condition. * and ** indicate significant differences at *p* < 0.05 and *p* < 0.01 per Student’s *t* test, respectively. Figure S7: Co-expression networks of the *BnaNF-Y* genes. Cycle nodes represent genes, and the size and color of the nodes represents the power of the interrelation among the nodes by degree value. The width and the color of the lines between two nodes represent interactions between two genes. The hub *NF-Y* genes are located in the center of the network, while the 10 most coexpressed genes are displayed in the network. Table S1: Statistics of the *NF-Y* genes in each subfamily in Arabidopsis and three *Brassica* species. Table S2: Copy number variations of the *NF-Y* family in higher plants. Table S3: Characteristics of the *NF-Y* family members in *B. napus* and subcellular localization prediction. Table S4: Non-synonymous (Ka) and synonymous (Ks) nucleotide substitution rates for the *NF-Y* coding loci in *Arabidopsis* and *B. napus*. Table S5. FPKM values of the *NF-Y* family genes in leaf and root of *B. napus* under N, P and K deficiencies based on the RNA-seq data. Table S6: Primers used for quantitative real-time PCR in this study.

## References

[1] Hahn S., Guarente L. (1988). Yeast HAP2 and HAP3: Transcriptional activators in a heteromeric complex. Science.

[2] Petroni K., Kumimoto R.W., Gnesutta N., Calvenzani V., Fornari M., Tonelli C., Holt B.F., Mantovani R. (2013). The Promiscuous Life of Plant NUCLEAR FACTOR Y Transcription Factors. Plant Cell.

[3] Laloum T., De Mita S., Gamas P., Baudin M., Niebel A. (2013). CCAAT-box binding transcription factors in plants: Y so many?. Trends Plant Sci..

[4] Myers Z.A., Holt B.F. (2018). NUCLEAR FACTOR-Y: Still complex after all these years?. Curr. Opin. Plant Biol..

[5] Siefers N., Dang K.K., Kumimoto R.W., Bynum W.E., Tayrose G., Holt B.F. (2009). Tissue-Specific Expression Patterns of Arabidopsis NF-Y Transcription Factors Suggest Potential for Extensive Combinatorial Complexity. Plant Physiol..

[6] Thirumurugan T., Ito Y., Kubo T., Serizawa A., Kurata N. (2008). Identification, characterization and interaction of HAP family genes in rice. Mol. Genet. Genom..

[7] Quach T.N., Nguyen H.T.M., Valliyodan B., Joshi T., Xu D., Nguyen H.T. (2015). Genome-wide expression analysis of soybean NF-Y genes reveals potential function in development and drought response. Mol. Genet. Genom..

[8] Li S., Li K., Ju Z., Cao D., Fu D., Zhu H., Zhu B., Luo Y. (2016). Genome-wide analysis of tomato NF-Y factors and their role in fruit ripening. BMC Genom..

[9] Zhang Z., Li X., Zhang C., Zou H., Wu Z. (2016). Isolation, structural analysis, and expression characteristics of the maize nuclear factor Y gene families. Biochem. Biophys. Res. Commun..

[10] Malviya N., Jaiswal P., Yadav D. (2016). Genome- wide characterization of Nuclear Factor Y (NF-Y) gene family of sorghum [Sorghum bicolor (L.) Moench]: A bioinformatics approach. Physiol. Mol. Biol. Plants.

[11] Maheshwari P., Kummari D., Palakolanu S.R., Tejaswi U.N., Nagaraju M., Rajasheker G., Jawahar G., Jalaja N., Rathnagiri P., Kishor P.B.K. (2019). Genome-wide identification and expression profile analysis of nuclear factor Y family genes in *Sorghum bicolor* L. (Moench). PLoS ONE.

[12] Pereira S.L.S., Martins C.P.S., Sousa A.O., Camillo L.R., Araújo C.P., Alcantara G.M., Camargo D.S., Cidade L.C., De Almeida A.-A.F., Costa M.G.C. (2018). Genome-wide characterization and expression analysis of citrus NUCLEAR FACTOR-Y (NF-Y) transcription factors identified a novel NF-YA gene involved in drought-stress response and tolerance. PLoS ONE.

[13] Panahi B., Mohammadi S.A., Ruzicka K., Holaso H.A., Mehrjerdi M.Z. (2019). Genome-wide identification and co-expression network analysis of nuclear factor-Y in barley revealed potential functions in salt stress. Physiol. Mol. Biol. Plants.

[14] Chen L., Zhou Y., Lai W., Hu L., Jiang L., Liu S. (2020). In Silico Identification and Expression Analysis of Nuclear Factor Y (Nf-Y) Transcription Factors in Cucumber. Agronomy.

[15] Qu Y., Wang Y., Zhu J., Zhang Y., Hou H. (2021). Genomic Organization, Phylogenetic Comparison, and Differential Expression of the Nuclear Factor-Y Gene Family in Apple (*Malus Domestica*). Plants.

[16] Chaves-Sanjuan A., Gnesutta N., Gobbini A., Martignago D., Bernardini A., Fornara F., Mantovani R., Nardini M. (2021). Structural determinants for NF-Y subunit organization and NF-Y/DNA association in plants. Plant J..

[17] Liu X., Hu P., Huang M., Tang Y., Li Y., Li L., Hou X. (2016). The NF-YC–RGL2 module integrates GA and ABA signalling to regulate seed germination in Arabidopsis. Nat. Commun..

[18] Wenkel S., Turck F., Singer K., Gissot L., Le Gourrierec J., Samach A., Coupland G. (2006). CONSTANS and the CCAAT Box Binding Complex Share a Functionally Important Domain and Interact to Regulate Flowering of Arabidopsis. Plant Cell.

[19] Cai X., Ballif J., Endo S., Davis E., Liang M., Chen D., DeWald D., Kreps J., Zhu T., Wu Y. (2007). A Putative CCAAT-Binding Transcription Factor Is a Regulator of Flowering Timing in Arabidopsis. Plant Physiol..

[20] Kumimoto R.W., Zhang Y., Siefers N., Holt B.F. (2010). NF-YC3, NF-YC4 and NF-YC9 are required for CONSTANS-mediated, photoperiod-dependent flowering in Arabidopsis thaliana. Plant J..

[21] Sorin C., Declerck M., Christ A., Blein T., Ma L., Lelandais-Brière C., Njo M.F., Beeckman T., Crespi M., Hartmann C. (2014). A miR 169 isoform regulates specific NF-YA targets and root architecture in Arabidopsis. New Phytol..

[22] Braybrook S., Harada J.J. (2008). LECs go crazy in embryo development. Trends Plant Sci..

[23] Xuanyuan G., Lu C., Zhang R., Jiang J. (2017). Overexpression of StNF-YB3.1 reduces photosynthetic capacity and tuber production, and promotes ABA-mediated stomatal closure in potato (Solanum tuberosum L.). Plant Sci..

[24] Miyoshi K., Ito Y., Serizawa A., Kurata N. (2003). OsHAP3genes regulate chloroplast biogenesis in rice. Plant J..

[25] Gago C., Drosou V., Paschalidis K., Guerreiro A., Miguel G., Antunes D., Hilioti Z. (2017). Targeted gene disruption coupled with metabolic screen approach to uncover the LEAFY COTYLEDON1-LIKE4 (L1L4) function in tomato fruit metabolism. Plant Cell Rep..

[26] Chen M., Zhao Y., Zhuo C., Lu S., Guo Z. (2015). Overexpression of aNF-YC transcription factor from bermudagrass confers tolerance to drought and salinity in transgenic rice. Plant Biotechnol. J..

[27] Hackenberg D., Keetman U., Grimm B. (2012). Homologous NF-YC2 Subunit from Arabidopsis and Tobacco Is Activated by Photooxidative Stress and Induces Flowering. Int. J. Mol. Sci..

[28] Nelson D.E., Repetti P.P., Adams T.R., Creelman R.A., Wu J., Warner D.C., Anstrom D.C., Bensen R.J., Castiglioni P.P., Donnarummo M.G. (2007). Plant nuclear factor Y (NF-Y) B subunits confer drought tolerance and lead to improved corn yields on water-limited acres. Proc. Natl. Acad. Sci. USA.

[29] Li W.-X., Oono Y., Zhu J., He X.-J., Wu J., Iida K., Lu X.-Y., Cui X., Jin H., Zhu J.-K. (2008). The Arabidopsis NFYA5 Transcription Factor Is Regulated Transcriptionally and Posttranscriptionally to Promote Drought Resistance. Plant Cell.

[30] Sato H., Suzuki T., Takahashi F., Shinozaki K., Yamaguchi-Shinozaki K. (2019). NF-YB2 and NF-YB3 Have Functionally Diverged and Differentially Induce Drought and Heat Stress-Specific Genes. Plant Physiol..

[31] Lee D.-K., Kim H.I., Jang G., Chung P.J., Jeong J.S., Kim Y.S., Bang S.W., Jung H., Choi Y.D., Kim J.-K. (2015). The NF-YA transcription factor OsNF-YA7 confers drought stress tolerance of rice in an abscisic acid independent manner. Plant Sci..

[32] Yang M., Zhao Y., Shi S., Du X., Gu J., Xiao K. (2017). Wheat nuclear factor Y (NF-Y) B subfamily gene TaNF-YB3;l confers critical drought tolerance through modulation of the ABA-associated signaling pathway. Plant Cell Tissue Organ Cult..

[33] Müller R., Morant M., Jarmer H., Nilsson L., Nielsen T.H. (2007). Genome-Wide Analysis of the Arabidopsis Leaf Transcriptome Reveals Interaction of Phosphate and Sugar Metabolism. Plant Physiol..

[34] Zanetti M.E., Rípodas C., Niebel A. (2017). Plant NF-Y transcription factors: Key players in plant-microbe interactions, root development and adaptation to stress. Biochim. Biophys. Acta.

[35] González M.A.L., Ibarra-Laclette E., Cruz-Ramírez L.A., Herrera-Estrella L. (2012). Functional and Transcriptome Analysis Reveals an Acclimatization Strategy for Abiotic Stress Tolerance Mediated by Arabidopsis NF-YA Family Members. PLoS ONE.

[36] Zhao M., Ding H., Zhu J.-K., Zhang F., Li W. (2011). Involvement of miR169 in the nitrogen-starvation responses in Arabidopsis. New Phytol..

[37] Qu B., He X., Wang J., Zhao Y., Teng W., Shao A., Zhao X., Ma W., Wang J., Li B. (2015). A Wheat CCAAT Box-Binding Transcription Factor Increases the Grain Yield of Wheat with Less Fertilizer Input. Plant Physiol..

[38] Lohani N., Jain D., Singh M.B., Bhalla P.L. (2020). Engineering Multiple Abiotic Stress Tolerance in Canola, *Brassica napus*. Front. Plant Sci..

[39] Snowdon R.J., Friedrich T., Friedt W., Köhler W. (2002). Identifying the chromosomes of the A- and C-genome diploid Brassica species B. rapa (syn. campestris) and B. oleracea in their amphidiploid B. napus. Theor. Appl. Genet..

[40] Chalhoub B., Denoeud F., Liu S., Parkin I.A.P., Tang H., Wang X., Chiquet J., Belcram H., Tong C., Samans B. (2014). Early allopolyploid evolution in the post-Neolithic Brassica napus oilseed genome. Science.

[41] Liu R., Wu M., Liu H., Gao Y., Chen J., Yan H., Xiang Y. (2021). Genome-wide identification and expression analysis of the NF-Y transcription factor family in Populus. Physiol. Plant..

[42] Liang M., Yin X., Lin Z., Zheng Q., Liu G., Zhao G. (2014). Identification and characterization of NF-Y transcription factor families in Canola (Brassica napus L.). Planta.

[43] Wang J., Jin Z., Zhou M., Yu Y., Liang M. (2020). Characterization of NF-Y transcription factor families in industrial rapeseed (*Brassica napus* L.) and identification of BnNF-YA3, which functions in the abiotic stress response. Ind. Crop. Prod..

[44] Wang P., Zheng Y., Guo Y., Chen X., Sun Y., Yang J., Ye N. (2019). Identification, expression, and putative target gene analysis of nuclear factor-Y (NF-Y) transcription factors in tea plant (Camellia sinensis). Planta.

[45] Ma X.-J., Yu T.-F., Li X.H., Cao X.-Y., Ma J., Chen J., Zhou Y.-B., Chen M., Ma Y.-Z., Zhang J.H. (2020). Overexpression of GmNFYA5 confers drought tolerance to transgenic Arabidopsis and soybean plants. BMC Plant Biol..

[46] Yu Y., Bai Y., Wang P., Wang Y., Wan H., Liu C., Ni Z. (2020). Soybean nuclear factor YA10 positively regulates drought resistance in transgenic Arabidopsis thaliana. Environ. Exp. Bot..

[47] Zhou Y., Zhang Y., Wang X., Han X., An Y., Lin S., Shen C., Wen J., Liu C., Yin W. (2020). Root-specific NF-Y family transcription factor, PdNF-YB21, positively regulates root growth and drought resistance by abscisic acid-mediated indoylacetic acid transport in Populus. New Phytol..

[48] Yang W., Lu Z., Xiong Y., Yao J. (2017). Genome-wide identification and co-expression network analysis of the OsNF-Y gene family in rice. Crop. J..

[49] Li L., Zheng W., Zhu Y., Ye H., Tang B., Arendsee Z.W., Jones D., Li R., Ortiz D., Zhao X. (2015). QQS orphan gene regulates carbon and nitrogen partitioning across species via NF-YC interactions. Proc. Natl. Acad. Sci. USA.

[50] Feng Z.-J., He G.-H., Zheng W.-J., Lu P.-P., Chen M., Gong Y.-M., Ma Y.-Z., Xu Z.-S. (2015). Foxtail Millet NF-Y Families: Genome-Wide Survey and Evolution Analyses Identified Two Functional Genes Important in Abiotic Stresses. Front. Plant Sci..

[51] Chen C., Chen H., Zhang Y., Thomas H.R., Frank M.H., He Y., Xia R. (2020). TBtools: An Integrative Toolkit Developed for Interactive Analyses of Big Biological Data. Mol. Plant.

[52] Wang Y., Tang H., DeBarry J., Tan X., Li J., Wang X., Lee T.-H., Jin H., Marler B., Guo H. (2012). MCScanX: A toolkit for detection and evolutionary analysis of gene synteny and collinearity. Nucleic Acids Res..

[53] Kumar S., Stecher G., Tamura K. (2016). MEGA7: Molecular Evolutionary Genetics Analysis Version 7.0 for Bigger Datasets. Mol. Biol. Evol..

[54] Nekrutenko A., Makova K.D., Li W.-H. (2002). The KA/KS Ratio Test for Assessing the Protein-Coding Potential of Genomic Regions: An Empirical and Simulation Study. Genome Res..

[55] Bailey T.L., Boden M., Buske F.A., Frith M., Grant C.E., Clementi L., Ren J., Li W.W., Noble W.S. (2009). MEME SUITE: Tools for motif discovery and searching. Nucleic Acids Res..

[56] Su G., Morris J.H., Demchak B., Bader G. (2014). Biological Network Exploration with Cytoscape 3. Curr. Protoc. Bioinform..

[57] Livak K.J., Schmittgen T.D. (2001). Analysis of relative gene expression data using real-time quantitative PCR and the 2(-Delta Delta C(T)) Method. Methods.

[58] Zhang H., Li S., Shi M., Wang S., Shi L., Xu F., Ding G. (2020). Genome-Wide Systematic Characterization of the *NPF* Family Genes and Their Transcriptional Responses to Multiple Nutrient Stresses in Allotetraploid Rapeseed. Int. J. Mol. Sci..

[59] Zhong C., Tang Y., Pang B., Li X., Yang Y., Deng J., Feng C., Li L., Ren G., Wang Y. (2020). The R2R3-MYB transcription factor GhMYB1a regulates flavonol and anthocyanin accumulation in Gerbera hybrida. Hortic. Res..

