# Supplementary material for: Identification and Comprehensive Analysis of the Nuclear Factor-Y Family Genes Reveal Their Multiple Roles in Response to Nutrient Deficiencies in Brassica napus
Source: Int J Mol Sci. 2021 Sep 26;22(19):10354. doi: 10.3390/ijms221910354 (PMC8508618; doi:10.3390/ijms221910354)
Supplement: Supplementary file 1 [file ijms-22-10354-s001.zip › ijms-1333611-supplementary/Figure S1-S7.pptx]

## Slide 1
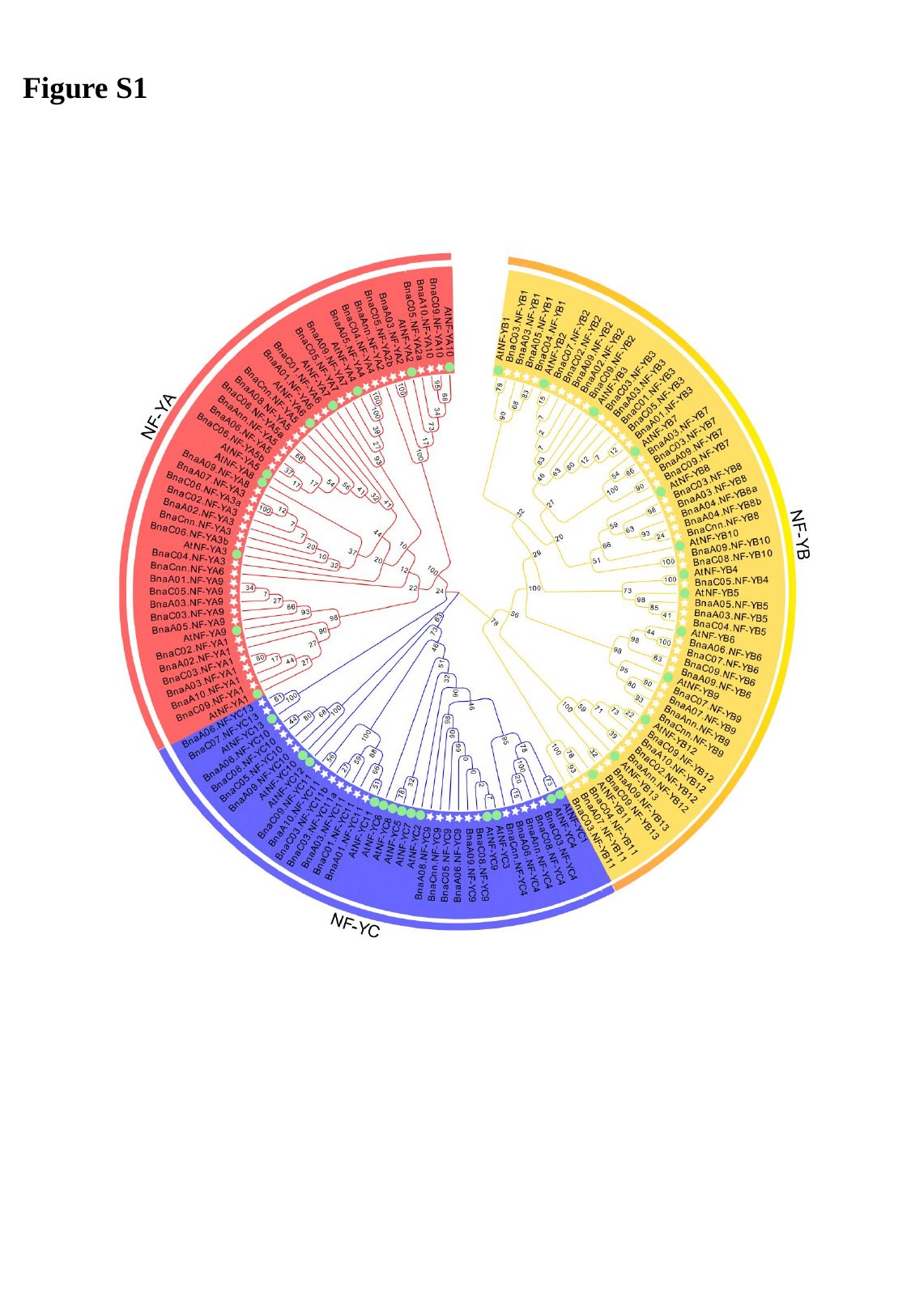

Figure S1

## Slide 2
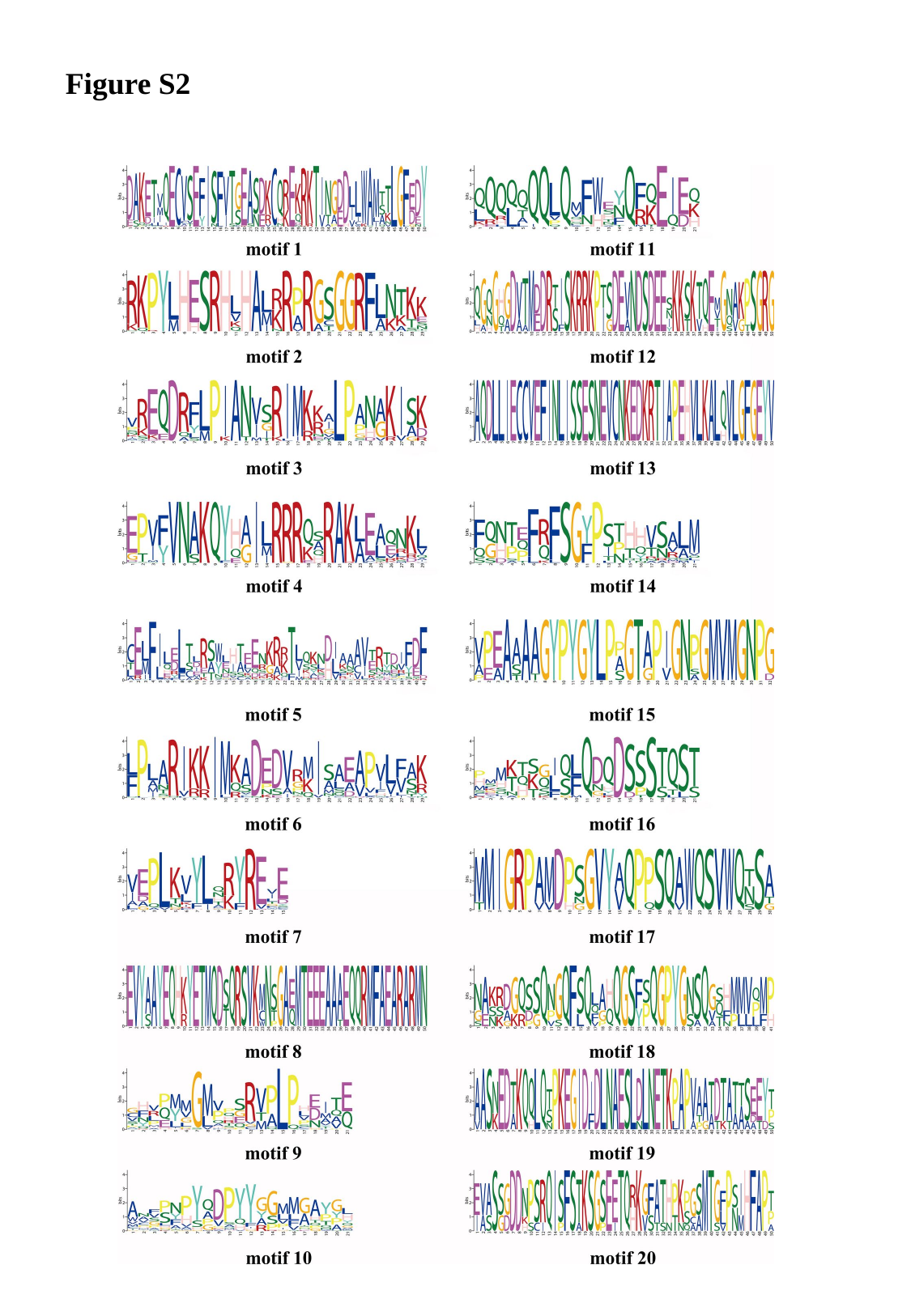

Figure S2

## Slide 3
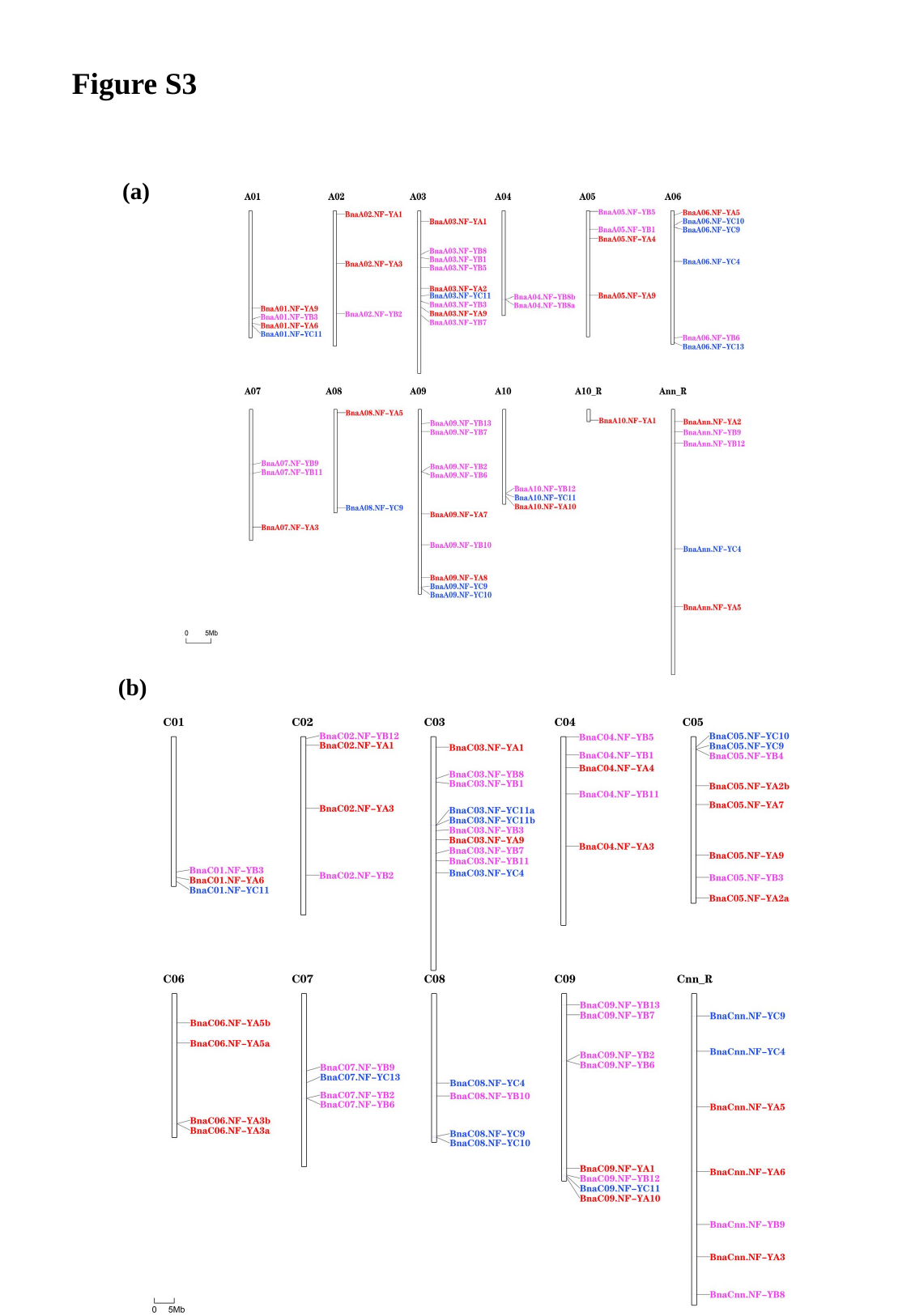

Figure S3
(a)
(b)

## Slide 4
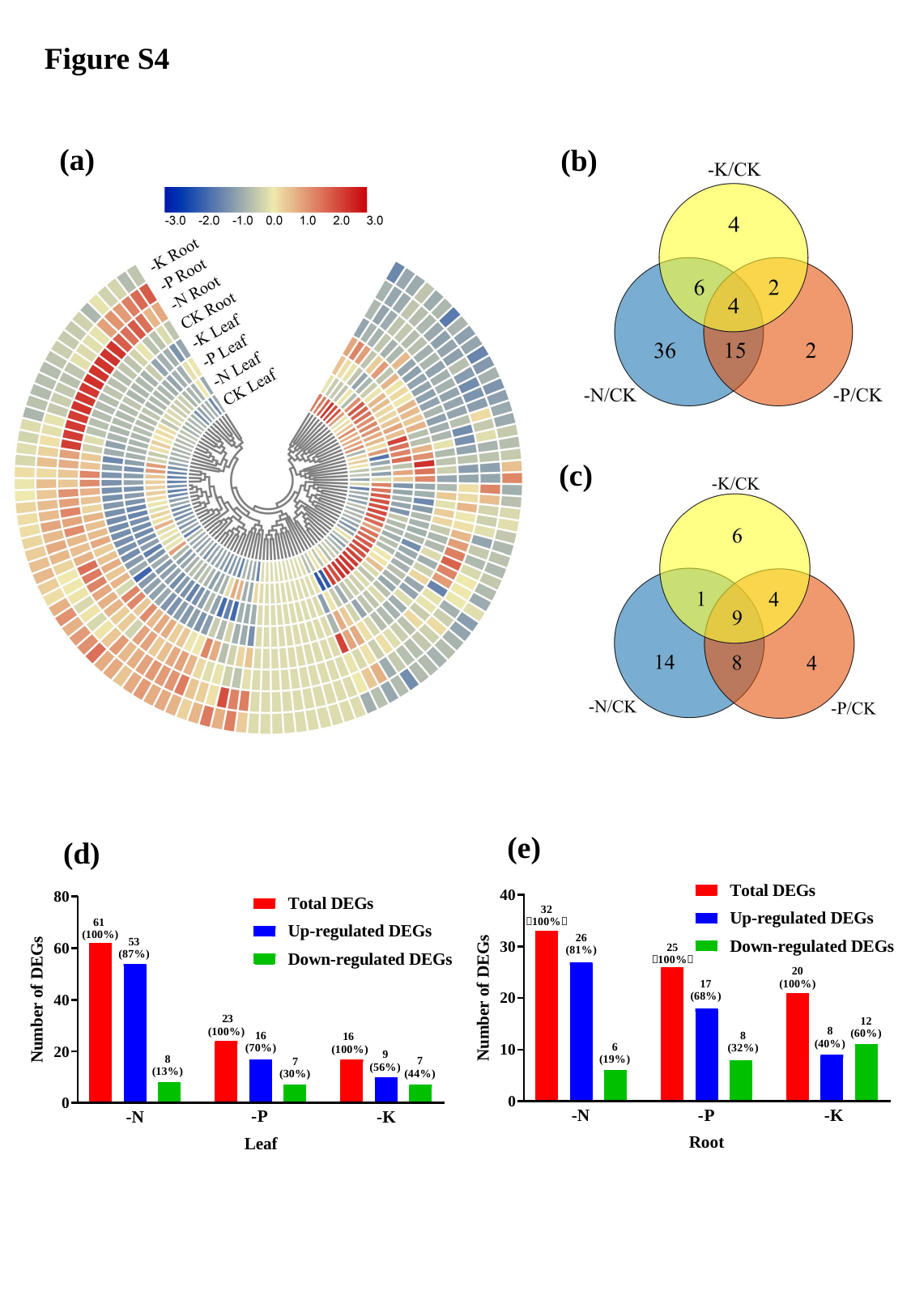

Figure S4
(a)
(b)
(c)
(e)
(d)

## Slide 5
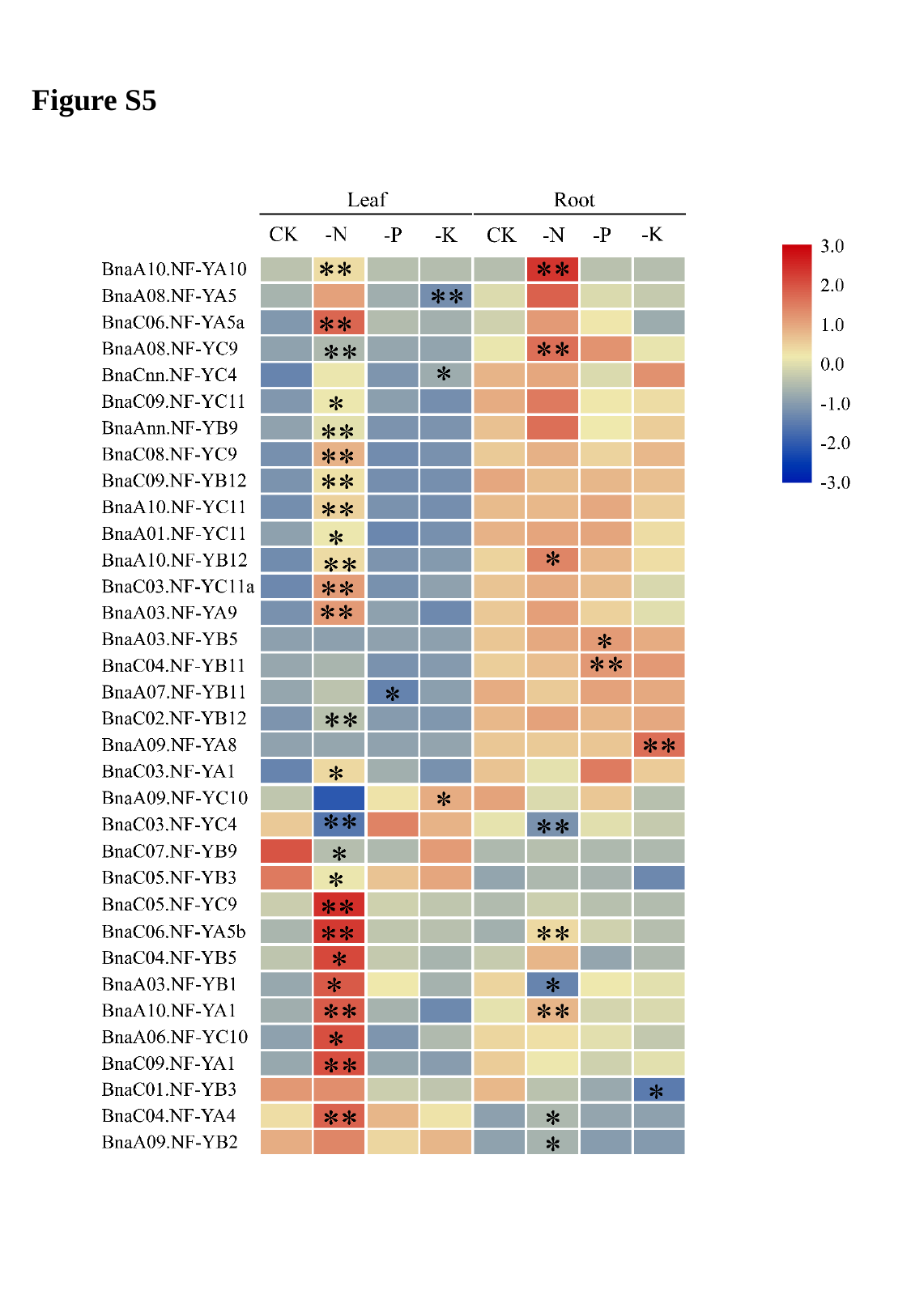

Figure S5

## Slide 6
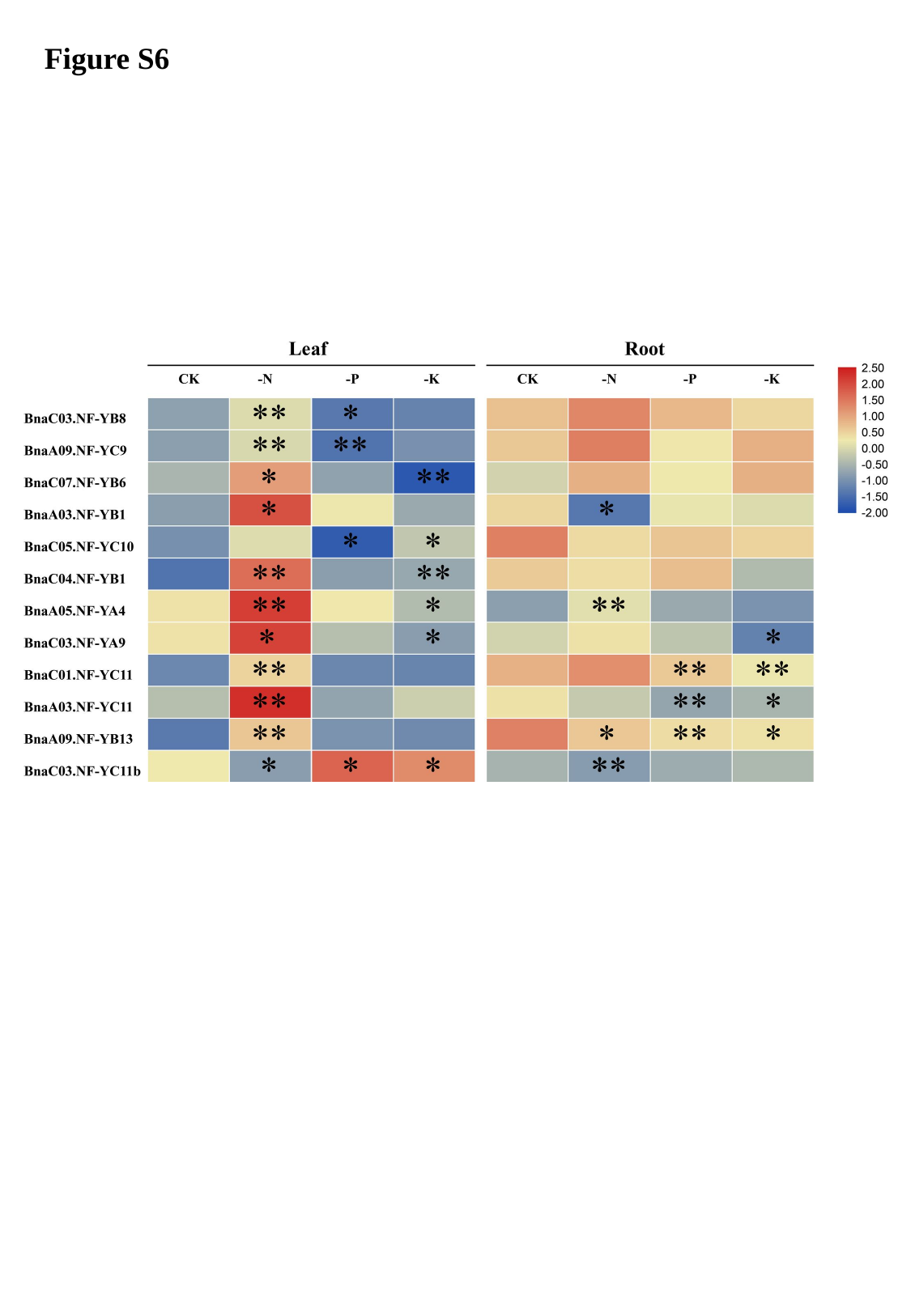

Figure S6

## Slide 7
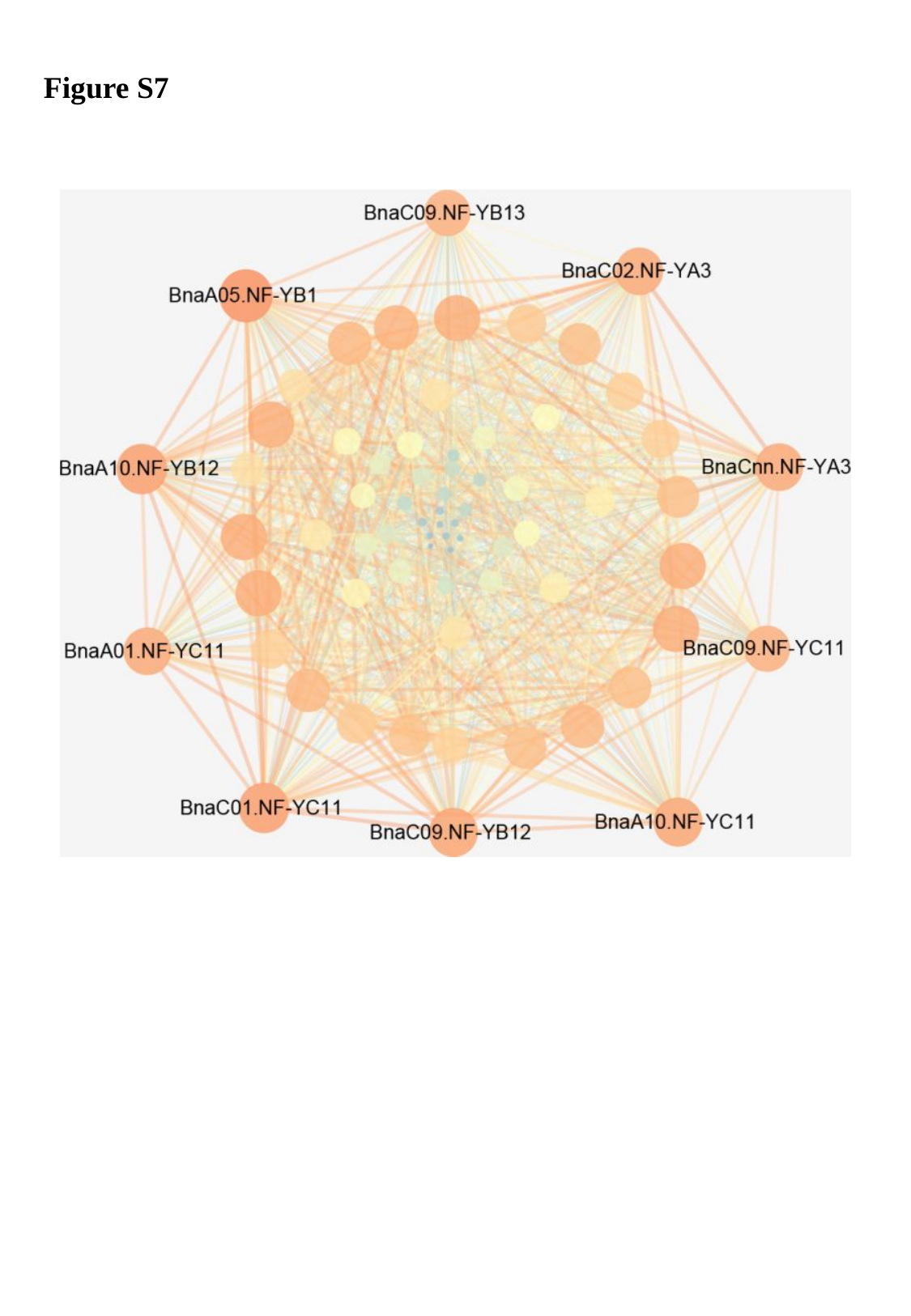

Figure S7
