# Supplementary material for: Identification and Comprehensive Analysis of the Nuclear Factor-Y Family Genes Reveal Their Multiple Roles in Response to Nutrient Deficiencies in Brassica napus
Source: Int J Mol Sci. 2021 Sep 26;22(19):10354. doi: 10.3390/ijms221910354 (PMC8508618; doi:10.3390/ijms221910354)
Supplement: Supplementary file 1 [file ijms-22-10354-s001.zip › ijms-1333611-supplementary/Table S1-S6.pdf]

Table S1. Statistics of the *NF-Y* genes in each *NF-Y* subfamily in *Arabidopsis* and three *Brassica* species

Type	Family	*Arabidopsis thaliana*	*Brassica oleracea*	*Brassica rapa*	*Brassica napus*
NF-YA	NF-YA1	1	3	3	6
	NF-YA2	1	3	2	4
	NF-YA3	1	7	2	7
	NF-YA4	1	1	1	2
	NF-YA5	1	2	3	6
	NF-YA6	1	2	1	3
	NF-YA7	1	1	1	2
	NF-YA8	1	1	1	1
	NF-YA9	1	1	3	5
	NF-YA10	1	1	1	2
NF-YB	NF-YB1	1	2	1	4
	NF-YB2	1	2	2	5
	NF-YB3	1	3	3	5
	NF-YB4	1	1	1	1
	NF-YB5	1	2	2	3
	NF-YB6	1	2	2	4
	NF-YB7	1	2	2	4
	NF-YB8	1	2	2	5
	NF-YB9	1	3	3	4
	NF-YB10	1	1	1	2
	NF-YB11	1	1	1	3
	NF-YB12	1	4	2	4
	NF-YB13	1	2	2	2
NF-YC	NF-YC1	1	/	/	/
	NF-YC2	1	1	1	/
	NF-YC3	1	/	/	/
	NF-YC4	1	3	2	5
	NF-YC5	1	/	/	/
	NF-YC6	1	/	/	/
	NF-YC7	1	/	/	/
	NF-YC8	1	/	/	/
	NF-YC9	1	2	3	6
	NF-YC10	1	2	2	4
	NF-YC11	1	3	3	7
	NF-YC12	1	1	/	/
	NF-YC13	1	1	1	2
Total	36	36	62	54	108

Table S2. Copy number variations of the *NF-Y* family in higher plants

Item	NF-YA	NF-YB	NF-YC	Total	Reference
Arabidopsis thaliana	10	13	13	36	TAIR database
Brassica napus	38	46	24	108	BLAST search in genome database
Brassica oleracea	22	27	13	62	BLAST search in genome database
Brassica rapa	18	24	12	54	BLAST search in genome database
Camellia sinensis	10	16	13	39	Planta. 2019, 250(5): 1671-1686
Glycine max	21	32	15	68	Mol Genetics Genomics. 2015, 290(3): 1095-1115
Juglans regia	17	9	7	33	BMC Plant Biology. 2018, 18(1): 255
Malus Domestica	11	22	10	43	Plants-Basel. 2020, 10(1): 16
Manihot esculenta	15	21	15	51	Annals of Botany. 2020, 124(7): 1185-1197
Petunia hybrida	10	13	4	27	Plants-Basel. 2020, 9(3): 336
Populus trichocarpa	13	20	19	52	Physiologia plantarum. 2021, 171(3): 309-327
Prunus persica	6	12	6	24	BMC Genomics. 2019, 20(1): 612
Sorghum bicolor	8	11	14	33	Physiol Mol Biol Plants. 2016, 22(1): 33-49
Triticum aestivum	10	11	14	35	Plant Mol Biol. 2007, 65(1-2): 77-92

Table S3. Characteristics of the *NF-Y* family members in *B. napus* and subcellular localization prediction

Gene name	Gene ID	Gene position			Gene length （bp）	Protein length （aa）	MW（Da）	PI	GRAVY	Intron number	Subcellular location
		Chrom.	Start	End							
BnaA02.NF-YA1	BnaA02g01270D	A2	566388	567760	1004	236	26098.88	6.67	-1.060	4	nucl
BnaA03.NF-YA1	BnaA03g04040D	A3	1883229	1884845	799	205	22509.00	8.58	-0.903	5	nucl
BnaA10.NF-YA1	BnaA10g29990D	A10_R	2015895	2018378	1655	284	30815.79	6.35	-1.018	4	nucl
BnaC02.NF-YA1	BnaC02g04360D	C2	2225135	2226235	660	219	24700.16	9.63	-0.629	5	nucl
BnaC03.NF-YA1	BnaC03g05640D	C3	2717638	2719734	1522	259	28567.61	8.52	-0.983	4	nucl
BnaC09.NF-YA1	BnaC09g44190D	C9	45143706	45146146	1645	285	31064.05	6.11	-1.019	4	nucl
BnaA03.NF-YA2	BnaA03g29140D	A3	14170614	14171911	846	281	30995.03	9.75	-0.625	3	nucl
BnaAnn.NF-YA2	BnaAnng02740D	An_R	2235220	2236987	1105	274	29736.43	9.39	-0.511	3	nucl
BnaC05.NF-YA2a	BnaC05g46110D	C5	41750761	41751533	540	179	20294.87	9.51	-0.777	3	nucl
BnaC05.NF-YA2b	BnaC05g19190D	C5	12704323	12706125	1351	281	30995.03	9.75	-0.625	3	nucl
BnaA02.NF-YA3	BnaA02g16180D	A2	9628040	9630116	1742	291	32281.88	8.89	-0.842	3	nucl
BnaA07.NF-YA3	BnaA07g30460D	A7	21566941	21569044	1707	322	35644.00	9.36	-0.707	4	nucl
BnaC02.NF-YA3	BnaC02g21570D	C2	18560619	18562639	1686	319	35316.44	9.03	-0.742	3	nucl
BnaC04.NF-YA3	BnaC04g27090D	C4	28335962	28336402	441	146	16422.19	8.58	0.027	0	nucl
BnaC06.NF-YA3a	BnaC06g33980D	C6	33618683	33620678	1032	343	38150.04	9.37	-0.663	5	nucl
BnaC06.NF-YA3b	BnaC06g33950D	C6	33612111	33612907	723	240	27265.27	9.72	0.023	1	chlo
BnaCnn.NF-YA3	BnaCnng68450D	Cn_R	68066532	68068714	1372	405	46094.97	9.73	-0.788	5	nucl
BnaA05.NF-YA4	BnaA05g09110D	A5	5018220	5020045	1152	197	22197.77	8.72	-1.024	4	nucl
BnaC04.NF-YA4	BnaC04g10470D	C4	8048154	8050217	1404	196	22080.65	8.93	-1.019	4	nucl
BnaA06.NF-YA5	BnaA06g01280D	A6	817024	818933	1655	285	31425.95	9.58	-0.769	3	nucl
BnaA08.NF-YA5	BnaA08g00840D	A8	610760	612272	1236	228	25409.68	9.45	-0.613	3	nucl
BnaAnn.NF-YA5	BnaAnng31560D	An_R	36113417	36115159	1470	231	25671.99	9.90	-0.670	3	nucl

BnaC06.NF-YA5a	BnaC06g10580D	C6	12712609	12714019	1157	235	25964.24	9.81	-0.640	3	nucl
BnaC06.NF-YA5b	BnaC06g06950D	C6	7497997	7499985	1772	299	32869.73	9.39	-0.626	4	nucl
BnaCnn.NF-YA5	BnaCnng30790D	Cn_R	29265164	29266560	1170	234	26028.40	9.61	-0.669	3	nucl
BnaA01.NF-YA6	BnaA01g29650D	A1	20492147	20493570	1034	308	33768.87	9.83	-0.638	4	nucl
BnaC01.NF-YA6	BnaC01g37260D	C1	36403731	36405134	1034	308	33961.12	9.73	-0.632	4	nucl
BnaCnn.NF-YA6	BnaCnng46640D	Cn_R	46011390	46011869	324	107	12211.62	5.56	-0.487	1	nucl
BnaA09.NF-YA7	BnaA09g26040D	A9	19158540	19160295	961	183	20800.31	9.04	-1.033	4	nucl
BnaC05.NF-YA7	BnaC05g23480D	C5	17579844	17581479	843	183	20802.31	9.06	-1.007	4	nucl
BnaA09.NF-YA8	BnaA09g44920D	A9	30778841	30780472	1186	322	35799.66	9.02	-0.856	4	nucl
BnaA01.NF-YA9	BnaA01g25500D	A1	17797274	17799488	763	180	20509.28	10.55	-1.228	2	nucl
BnaA03.NF-YA9	BnaA03g35980D	A3	17592029	17593794	1350	280	31103.08	8.97	-1.185	4	nucl
BnaA05.NF-YA9	BnaA05g19990D	A5	15425888	15427902	1478	294	32395.42	6.36	-1.090	4	nucl
BnaC03.NF-YA9	BnaC03g41820D	C3	26687276	26689145	1442	298	32927.06	8.92	-1.139	4	nucl
BnaC05.NF-YA9	BnaC05g31370D	C5	30686248	30688424	1605	298	32912.16	8.84	-1.107	4	nucl
BnaA10.NF-YA10	BnaA10g24470D	A10	15949384	15950501	853	267	29845.72	9.46	-0.675	3	nucl
BnaC09.NF-YA10	BnaC09g49050D	C9	47617066	47618130	804	267	29624.62	9.42	-0.578	3	nucl
BnaA03.NF-YB1	BnaA03g18200D	A3	8535232	8536613	756	166	18111.55	5.52	-0.331	2	nucl
BnaA05.NF-YB1	BnaA05g06260D	A5	3332868	3334807	1341	142	15283.00	4.95	-0.683	2	nucl
BnaC03.NF-YB1	BnaC03g21730D	C3	11759347	11760703	750	164	18071.54	5.23	-0.201	2	nucl
BnaC04.NF-YB1	BnaC04g06490D	C4	4728865	4731076	912	212	23063.97	8.09	-0.570	4	nucl
BnaA02.NF-YB2	BnaA02g25600D	A2	18785085	18785860	776	186	19901.88	5.96	-0.885	0	nucl
BnaA09.NF-YB2	BnaA09g18400D	A9	11446337	11447022	686	184	19883.80	9.96	-0.962	0	nucl
BnaC02.NF-YB2	BnaC02g33400D	C2	35853002	35853683	682	185	19967.00	6.30	-0.894	0	nucl
BnaC07.NF-YB2	BnaC07g20390D	C7	27011240	27011748	405	134	14620.21	4.80	-0.640	1	chlo
BnaC09.NF-YB2	BnaC09g20320D	C9	17368804	17369486	683	187	20177.12	6.07	-0.943	0	nucl
BnaA01.NF-YB3	BnaA01g28350D	A1	19803940	19804416	477	158	17024.90	6.06	-0.773	0	nucl

BnaA03.NF-YB3	BnaA03g33970D	A3	16504578	16505412	835	189	19644.46	6.16	-0.755	0	nucl
BnaC01.NF-YB3	BnaC01g35600D	C1	35046879	35047591	713	157	16829.72	6.21	-0.738	0	nucl
BnaC03.NF-YB3	BnaC03g39380D	C3	24331939	24332773	835	186	19548.41	6.30	-0.796	0	nucl
BnaC05.NF-YB3	BnaC05g37390D	C5	36433213	36433710	498	165	17461.26	5.95	-0.756	0	nucl
BnaC05.NF-YB4	BnaC05g06570D	C5	3248229	3248627	399	132	14857.47	6.15	-0.831	0	mito
BnaA03.NF-YB5	BnaA03g21860D	A3	10344600	10345070	471	156	17901.02	6.38	-0.900	0	nucl
BnaA05.NF-YB5	BnaA05g00200D	A5	100806	101457	519	172	19841.33	6.50	-0.783	1	nucl
BnaC04.NF-YB5	BnaC04g00090D	C4	31312	31998	687	155	17617.76	6.70	-0.852	0	nucl
BnaA06.NF-YB6	BnaA06g35340D	A6	23271611	23272448	696	231	25910.02	6.18	-0.723	1	nucl
BnaA09.NF-YB6	BnaA09g18420D	A9	11452614	11453343	665	209	23339.17	5.98	-0.666	1	nucl
BnaC07.NF-YB6	BnaC07g20410D	C7	27026011	27028908	1995	642	73177.87	7.61	-0.800	3	nucl
BnaC09.NF-YB6	BnaC09g20350D	C9	17373524	17374297	702	207	23164.04	5.98	-0.616	1	nucl
BnaA03.NF-YB7	BnaA03g38390D	A3	19081916	19082545	630	209	23901.25	5.84	-1.107	0	nucl
BnaA09.NF-YB7	BnaA09g08330D	A9	4084474	4085100	627	208	23718.17	5.84	-1.056	0	nucl
BnaC03.NF-YB7	BnaC03g45220D	C3	30228273	30228902	630	209	23897.26	5.84	-1.111	0	nucl
BnaC09.NF-YB7	BnaC09g08600D	C9	5489196	5489822	627	208	23791.22	5.58	-1.053	0	nucl
BnaA03.NF-YB8	BnaA03g16960D	A3	7957582	7959701	954	267	29515.26	8.80	-0.563	7	nucl
BnaA04.NF-YB8a	BnaA04g21270D	A4	16208767	16210237	585	194	21354.82	7.75	-0.634	3	nucl
BnaA04.NF-YB8b	BnaA04g21250D	A4	16196319	16197903	528	175	19276.37	6.42	-0.818	4	nucl
BnaC03.NF-YB8	BnaC03g20500D	C3	10865305	10867168	669	222	24355.50	8.98	-0.577	6	nucl
BnaCnn.NF-YB8	BnaCnng76740D	Cn_R	77820665	77822121	561	186	20667.95	6.97	-0.677	3	nucl
BnaA07.NF-YB9	BnaA07g10770D	A7	10129311	10131376	893	231	25381.37	5.96	-0.702	1	nucl
BnaAnn.NF-YB9	BnaAnng04140D	An_R	4158907	4163093	1283	342	37734.63	5.50	-0.391	2	plas
BnaC07.NF-YB9	BnaC07g14150D	C7	19943199	19945282	886	230	25358.34	5.96	-0.701	1	nucl
BnaCnn.NF-YB9	BnaCnng59920D	Cn_R	59697476	59698858	903	300	33181.05	5.39	-0.568	1	nucl
BnaA09.NF-YB10	BnaA09g33640D	A9	24770165	24772100	967	257	28388.05	9.44	-0.574	6	nucl

BnaC08.NF-YB10	BnaC08g24430D	C8	26421510	26423319	942	229	25235.36	9.62	-0.843	6	nucl
BnaA07.NF-YB11	BnaA07g13230D	A7	11774794	11775938	814	218	24038.64	4.34	-1.267	2	nucl
BnaC03.NF-YB11	BnaC03g47130D	C3	32107109	32108310	830	211	23501.18	4.42	-1.358	2	nucl
BnaC04.NF-YB11	BnaC04g16850D	C4	14822766	14823867	746	214	23519.08	4.36	-1.244	2	nucl
BnaA10.NF-YB12	BnaA10g23160D	A10	15378003	15379167	901	165	18428.65	4.57	-0.624	3	chlo
BnaAnn.NF-YB12	BnaAnng06230D	An_R	6195869	6197811	747	248	28398.43	6.72	-0.577	4	nucl
BnaC02.NF-YB12	BnaC02g01260D	C2	517056	519849	1629	240	27347.38	6.93	-0.523	5	cyto
BnaC09.NF-YB12	BnaC09g47720D	C9	46915815	46917083	964	166	18443.63	4.57	-0.651	3	chlo
BnaA09.NF-YB13	BnaA09g05430D	A9	2669540	2671009	1113	169	19047.31	4.74	-0.755	4	chlo
BnaC09.NF-YB13	BnaC09g05000D	C9	2888970	2890360	1061	169	19061.33	4.74	-0.754	4	chlo
BnaA06.NF-YC4	BnaA06g16420D	A6	9223546	9224734	1189	221	24244.41	5.06	-0.400	0	nucl
BnaAnn.NF-YC4	BnaAnng22700D	An_R	25505210	25506448	1113	273	29633.70	5.17	-0.285	3	chlo
BnaC03.NF-YC4	BnaC03g50810D	C3	35230481	35231749	1144	283	30821.17	5.54	-0.292	2	chlo
BnaC08.NF-YC4	BnaC08g20340D	C8	23076932	23078225	1294	221	24301.47	5.06	-0.414	0	nucl
BnaCnn.NF-YC4	BnaCnng15880D	Cn_R	14839773	14840940	1011	264	29559.47	5.09	-0.381	2	chlo
BnaA06.NF-YC9	BnaA06g05230D	A6	2982168	2983799	1400	220	24496.60	5.23	-0.535	1	nucl
BnaA08.NF-YC9	BnaA08g26690D	A8	17990349	17991630	1201	191	21299.78	5.13	-0.688	1	nucl
BnaA09.NF-YC9	BnaA09g48620D	A9	32533160	32534650	1491	228	25421.53	5.10	-0.665	0	nucl
BnaC05.NF-YC9	BnaC05g06520D	C5	3224198	3228539	2674	327	36931.74	7.08	-0.641	2	mito
BnaC08.NF-YC9	BnaC08g42880D	C8	36874233	36875771	1382	254	28661.58	5.75	-0.477	2	nucl
BnaCnn.NF-YC9	BnaCnng06510D	Cn_R	5706615	5711120	891	296	33273.15	9.44	-0.462	3	chlo
BnaA06.NF-YC10	BnaA06g04450D	A6	2644126	2645359	534	177	20110.71	8.51	-0.927	3	nucl
BnaA09.NF-YC10	BnaA09g49120D	A9	32763228	32764356	705	181	20346.87	9.04	-0.931	3	nucl
BnaC05.NF-YC10	BnaC05g05650D	C5	2744017	2745474	733	182	20606.17	9.04	-0.925	3	nucl
BnaC08.NF-YC10	BnaC08g43430D	C8	37158427	37160035	726	182	20412.79	8.87	-1.004	3	nucl
BnaA01.NF-YC11	BnaA01g30580D	A1	20947665	20949601	1079	289	31884.32	4.99	-0.936	5	chlo

BnaA03.NF-YC11	BnaA03g32260D	A3	15566754	15568973	1064	287	31999.61	5.02	-0.894	5	chlo
BnaA10.NF-YC11	BnaA10g23530D	A10	15532923	15534807	1135	299	33266.07	5.08	-0.892	5	chlo
BnaC01.NF-YC11	BnaC01g38370D	C1	37474332	37476339	1052	293	32473.95	4.96	-0.998	5	chlo
BnaC03.NF-YC11a	BnaC03g37550D	C3	22991247	22993437	1077	287	31947.45	5.00	-0.891	5	chlo
BnaC03.NF-YC11b	BnaC03g37560D	C3	22999722	23001891	1021	287	31876.33	4.95	-0.898	5	chlo
BnaC09.NF-YC11	BnaC09g48240D	C9	47154793	47156984	1330	302	33389.18	5.03	-0.853	5	chlo
BnaA06.NF-YC13	BnaA06g36810D	A6	24060533	24061095	563	130	14755.12	9.99	-0.589	0	nucl
BnaC07.NF-YC13	BnaC07g16980D	C7	22984339	22984728	390	129	14623.94	9.82	-0.518	0	cyto

Note: Chrom., Chromosome; MW, molecular weight; PI, isoelectric point; plas, plasma membrane; cyto, cytoplasm; nucl, nucleus;mito， mitochondrion; chlo, chloroplast membrane

Table S4. Non-synonymous (Ka) and synonymous (Ks) nucleotide substitution rates for the *NF-Y* coding loci in *Arabidopsis* and *B. napus*

Arabidopsis ID	*Arabidopsis* gene name	*B. napus* ID	*B. napus* gene name	Ka	Ks	Ka/Ks
AT5G12840	*AtNF-YA1*	BnaA02g01270D	*BnaA02.NF-YA1*	0.123206444	0.363923937	0.338549986
		BnaA03g04040D	*BnaA03.NF-YA1*	0.113379103	0.399024259	0.284140877
		BnaA10g29990D	*BnaA10.NF-YA1*	0.086137438	0.453682105	0.189862983
		BnaC02g04360D	*BnaC02.NF-YA1*	0.200575809	0.535990039	0.374215553
		BnaC03g05640D	*BnaC03.NF-YA1*	0.090648897	0.394859789	0.229572369
		BnaC09g44190D	*BnaC09.NF-YA1*	0.081562819	0.46978933	0.173615733
AT3G05690	*AtNF-YA2*	BnaA03g29140D	*BnaA03.NF-YA2*	0.089695461	0.236286725	0.379604318
		BnaAnng02740D	*BnaAnn.NF-YA2*	0.082756096	0.235848573	0.350886568
		BnaC05g46110D	*BnaC05.NF-YA2a*	0.110122404	0.432598157	0.254560502
		BnaC05g19190D	*BnaC05.NF-YA2b*	0.089695461	0.236286725	0.379604318
AT1G72830	*AtNF-YA3*	BnaA02g16180D	*BnaA02.NF-YA3*	0.092854126	0.381774624	0.243217123
		BnaA07g30460D	*BnaA07.NF-YA3*	0.096342883	0.397583178	0.242321327
		BnaC02g21570D	*BnaC02.NF-YA3*	0.057713923	0.396554157	0.145538565
		BnaC04g27090D	*BnaC04.NF-YA3*	0.568510165	1.027176765	0.553468677
		BnaCnng68450D	*BnaCnn.NF-YA3*	0.084005481	0.390937173	0.214882304
		BnaC06g33980D	*BnaC06.NF-YA3a*	0.122057951	0.407260383	0.299704945
		BnaC06g33950D	*BnaC06.NF-YA3b*	0.400651814	1.009180615	0.397007045
AT2G34720	*AtNF-YA4*	BnaA05g09110D	*BnaA05.NF-YA4*	0.055388482	0.35121232	0.157706547
		BnaC04g10470D	*BnaC04.NF-YA4*	0.057921477	0.347937887	0.166470737

AT1G54160	*AtNF-YA5*	BnaA06g01280D	*BnaA06.NF-YA5*	0.096088016	0.293035724	0.327905468
		BnaA08g00840D	*BnaA08.NF-YA5*	0.09892116	0.26773276	0.369477235
		BnaAnng31560D	*BnaAnn.NF-YA5*	0.11253172	0.363629865	0.309467759
		BnaCnng30790D	*BnaCnn.NF-YA5*	0.089497011	0.350047728	0.255670881
		BnaC06g10580D	*BnaC06.NF-YA5a*	0.115658252	0.334091219	0.346187644
		BnaC06g06950D	*BnaC06.NF-YA5b*	0.143846681	0.273085089	0.52674674
AT3G14020	*AtNF-YA6*	BnaA01g29650D	*BnaA01.NF-YA6*	0.135360039	0.407230764	0.332391486
		BnaC01g37260D	*BnaC01.NF-YA6*	0.161708692	0.44600353	0.362572672
		BnaCnng46640D	*BnaCnn.NF-YA6*	0.310501222	0.834205277	0.372212009
AT1G30500	*AtNF-YA7*	BnaA09g26040D	*BnaA09.NF-YA7*	0.095063779	0.392823605	0.242001189
		BnaC05g23480D	*BnaC05.NF-YA7*	0.100786029	0.408402511	0.246781119
AT1G17590	*AtNF-YA8*	BnaA09g44920D	*BnaA09.NF-YA8*	0.125409141	0.295604306	0.424246664
AT3G20910	*AtNF-YA9*	BnaA01g25500D	*BnaA01.NF-YA9*	0.166691594	0.323992561	0.514492043
		BnaA03g35980D	*BnaA03.NF-YA9*	0.104666588	0.310372326	0.337229126
		BnaA05g19990D	*BnaA05.NF-YA9*	0.128819569	0.312106595	0.412742222
		BnaC03g41820D	*BnaC03.NF-YA9*	0.133212103	0.291257099	0.457369462
		BnaC05g31370D	*BnaC05.NF-YA9*	0.103894636	0.319339745	0.325342014
AT5G06510	*AtNF-YA10*	BnaA10g24470D	*BnaA10.NF-YA10*	0.154110067	0.33217843	0.463937609
		BnaC09g49050D	*BnaC09.NF-YA10*	0.130075029	0.374068741	0.347730282
AT2G38880	*AtNF-YB1*	BnaA03g18200D	*BnaA03.NF-YB1*	0.066205861	0.642079383	0.103111645
		BnaA05g06260D	*BnaA05.NF-YB1*	0.026055595	0.699719294	0.037237211
		BnaC03g21730D	*BnaC03.NF-YB1*	0.127492277	0.776262735	0.164238564
		BnaC04g06490D	*BnaC04.NF-YB1*	0.028179678	0.60428387	0.046633179
AT5G47640	*AtNF-YB2*	BnaA02g25600D	*BnaA02.NF-YB2*	0.021341096	0.542844819	0.039313438
		BnaA09g18400D	*BnaA09.NF-YB2*	0.053140933	0.65883763	0.080658619

		BnaC02g33400D	*BnaC02.NF-YB2*	0.029914234	0.614717775	0.048663363
		BnaC07g20390D	*BnaC07.NF-YB2*	0.15305107	0.667759808	0.229200782
		BnaC09g20320D	*BnaC09.NF-YB2*	0.042945541	0.634800297	0.067652049
AT4G14540	*AtNF-YB3*	BnaA01g28350D	*BnaA01.NF-YB3*	0.071749967	2.096129498	0.03422974
		BnaA03g33970D	*BnaA03.NF-YB3*	0.095164179	1.082254924	0.087931389
		BnaC01g35600D	*BnaC01.NF-YB3*	0.075475652	1.693158276	0.044576844
		BnaC03g39380D	*BnaC03.NF-YB3*	0.109742558	1.279799715	0.085749791
		BnaC05g37390D	*BnaC05.NF-YB3*	0.102870693	1.302235919	0.078995435
AT1G09030	*AtNF-YB4*	BnaC05g06570D	*BnaC05.NF-YB4*	0.062840476	0.411313097	0.152780148
AT2G47810	*AtNF-YB5*	BnaA03g21860D	*BnaA03.NF-YB5*	0.067572049	0.333462599	0.202637565
		BnaA05g00200D	*BnaA05.NF-YB5*	0.071177129	0.435694181	0.163364883
		BnaC04g00090D	*BnaC04.NF-YB5*	0.060853731	0.424551608	0.143336476
AT5G47670	*AtNF-YB6*	BnaA06g35340D	*BnaA06.NF-YB6*	0.078734964	0.318895365	0.246899054
		BnaA09g18420D	*BnaA09.NF-YB6*	0.0860104	0.339749556	0.25315824
		BnaC07g20410D	*BnaC07.NF-YB6*	0.077979497	0.32319918	0.241273808
		BnaC09g20350D	*BnaC09.NF-YB6*	0.075377191	0.401508403	0.187735028
AT2G13570	*AtNF-YB7*	BnaA03g38390D	*BnaA03.NF-YB7*	0.060116495	0.58257099	0.103191707
		BnaA09g08330D	*BnaA09.NF-YB7*	0.092067404	0.561638115	0.163926559
		BnaC03g45220D	*BnaC03.NF-YB7*	0.057903174	0.566459221	0.102219492
		BnaC09g08600D	*BnaC09.NF-YB7*	0.096594591	0.514347497	0.187800255
AT2G37060	*AtNF-YB8*	BnaA03g16960D	*BnaA03.NF-YB8*	0.024195647	0.519860385	0.046542586
		BnaC03g20500D	*BnaC03.NF-YB8*	0.027181577	0.468270547	0.058046737
		BnaCnng76740D	*BnaCnn.NF-YB8*	0.112764055	0.667858351	0.168844268
		BnaA04g21270D	*BnaA04.NF-YB8a*	0.109969019	0.756128763	0.145436894
		BnaA04g21250D	*BnaA04.NF-YB8b*	0.055869978	0.600688936	0.093009834

AT1G21970	*AtNF-YB9*	BnaA07g10770D	*BnaA07.NF-YB9*	0.133651589	0.372078279	0.359202879
		BnaAnng04140D	*BnaAnn.NF-YB9*	0.141221022	0.451235599	0.31296516
		BnaC07g14150D	*BnaC07.NF-YB9*	0.13450359	0.39070561	0.34425815
		BnaCnng59920D	*BnaCnn.NF-YB9*	0.155006326	0.484280048	0.320075804
AT3G53340	*AtNF-YB10*	BnaA09g33640D	*BnaA09.NF-YB10*	0.067636791	0.64453695	0.104938578
		BnaC08g24430D	*BnaC08.NF-YB10*	0.064919396	0.583278426	0.11130087
AT2G27470	*AtNF-YB11*	BnaA07g13230D	*BnaA07.NF-YB11*	0.111953809	0.439657499	0.254638689
		BnaC03g47130D	*BnaC03.NF-YB11*	0.175594224	0.464327703	0.378168743
		BnaC04g16850D	*BnaC04.NF-YB11*	0.118420342	0.414308974	0.285826158
AT5G08190	*AtNF-YB12*	BnaA10g23160D	*BnaA10.NF-YB12*	0.052605826	0.766766884	0.068607327
		BnaAnng06230D	*BnaAnn.NF-YB12*	0.059478157	0.755103555	0.078768213
		BnaC02g01260D	*BnaC02.NF-YB12*	0.040909936	0.700346819	0.058413824
		BnaC09g47720D	*BnaC09.NF-YB12*	0.044497434	0.611055381	0.072820623
AT5G23090	*AtNF-YB13*	BnaA09g05430D	*BnaA09.NF-YB13*	0.024436551	0.43695398	0.05592477
		BnaC09g05000D	*BnaC09.NF-YB13*	0.024480868	0.383119218	0.063898825
AT5G63470	*AtNF-YC4*	BnaA06g16420D	*BnaA06.NF-YC4*	0.078420002	1.336788947	0.058662964
		BnaAnng22700D	*BnaAnn.NF-YC4*	0.071783343	0.866964626	0.082798468
		BnaC03g50810D	*BnaC03.NF-YC4*	0.072795672	0.919600095	0.07916014
		BnaC08g20340D	*BnaC08.NF-YC4*	0.072937439	1.25650267	0.058047978
		BnaCnng15880D	*BnaCnn.NF-YC4*	0.104856762	1.091525183	0.096064446
AT1G08970	*AtNF-YC9*	BnaA06g05230D	*BnaA06.NF-YC9*	0.064934937	0.772524486	0.084055507
		BnaA08g26690D	*BnaA08.NF-YC9*	0.065604843	0.5585245	0.117460995
		BnaA09g48620D	*BnaA09.NF-YC9*	0.022906186	0.928760126	0.024663189
		BnaC05g06520D	*BnaC05.NF-YC9*	0.040635441	0.657009939	0.061849051
		BnaC08g42880D	*BnaC08.NF-YC9*	0.096649615	1.33604327	0.072340183

		BnaCnng06510D	*BnaCnn.NF-YC9*	0.051065937	0.652484434	0.078263839
AT1G07980	*AtNF-YC10*	BnaA06g04450D	*BnaA06.NF-YC10*	0.124296411	0.569634163	0.218203926
		BnaA09g49120D	*BnaA09.NF-YC10*	0.134666801	0.520784828	0.25858434
		BnaC05g05650D	*BnaC05.NF-YC10*	0.138155856	0.799182189	0.172871541
		BnaC08g43430D	*BnaC08.NF-YC10*	0.104334602	0.505678145	0.206326105
AT3G12480	*AtNF-YC11*	BnaA01g30580D	*BnaA01.NF-YC11*	0.07596472	0.389199158	0.195182128
		BnaA03g32260D	*BnaA03.NF-YC11*	0.085501735	0.357857763	0.238926589
		BnaA10g23530D	*BnaA10.NF-YC11*	0.190063509	0.752055453	0.252725392
		BnaC01g38370D	*BnaC01.NF-YC11*	0.082479048	0.418247156	0.197201694
		BnaC09g48240D	*BnaC09.NF-YC11*	0.193291066	0.793276087	0.243661783
		BnaC03g37550D	*BnaC03.NF-YC11a*	0.083992688	0.380505975	0.220739473
		BnaC03g37560D	*BnaC03.NF-YC11b*	0.082274188	0.364130862	0.225946759
AT5G43250	*AtNF-YC13*	BnaA06g36810D	*BnaA06.NF-YC13*	0.132230004	0.789131531	0.167563959
		BnaC07g16980D	*BnaC07.NF-YC13*	0.099905629	0.807475036	0.123725967

Table S5. FPKM values of the *NF-Y* family genes in leaf and root of *B. napus* under N, P and K deficiencies based on the RNA-seq data

Gene name	Gene ID	Leaf_CK	Leaf_LN	Leaf_LP	Leaf_LK	Root_CK	Root_LN	Root_LP	Root_LK
BnaA02.NF-YA1	BnaA02g01270D	2.13	1.20	0.68	1.35	0.76	0.92	0.85	0.71
BnaA03.NF-YA1	BnaA03g04040D	0.06	0.00	0.00	0.00	0.00	0.00	0.00	0.00
BnaA10.NF-YA1	BnaA10g29990D	4.34	10.42	4.48	2.98	6.17	7.89	5.71	5.86
BnaC02.NF-YA1	BnaC02g04360D	0.37	0.52	0.62	0.17	0.14	0.00	0.20	0.10
BnaC03.NF-YA1	BnaC03g05640D	0.78	2.23	1.33	0.94	2.44	1.95	3.15	2.36
BnaC09.NF-YA1	BnaC09g44190D	4.49	8.78	4.44	4.23	6.54	5.95	5.43	5.78
BnaA03.NF-YA2	BnaA03g29140D	0.71	6.11	1.17	0.67	19.90	184.26	127.73	37.71
BnaAnn.NF-YA2	BnaAnng02740D	0.28	3.89	0.52	0.22	0.35	14.39	1.65	0.28
BnaC05.NF-YA2a	BnaC05g46110D	0.06	0.14	0.07	0.00	0.20	0.72	0.21	0.20
BnaC05.NF-YA2b	BnaC05g19190D	0.52	4.71	1.28	0.88	21.19	173.82	162.28	44.08
BnaA02.NF-YA3	BnaA02g16180D	0.40	3.30	0.72	0.61	1.09	16.56	3.41	2.12
BnaA07.NF-YA3	BnaA07g30460D	0.71	4.02	1.21	0.67	2.73	25.60	6.76	4.63
BnaC02.NF-YA3	BnaC02g21570D	2.05	10.29	4.15	3.47	10.49	40.67	16.23	10.70
BnaC04.NF-YA3	BnaC04g27090D	0.00	0.00	0.00	0.00	0.00	0.00	0.00	0.00
BnaC06.NF-YA3a	BnaC06g33980D	2.06	11.67	2.83	2.63	5.37	52.07	13.41	8.70
BnaC06.NF-YA3b	BnaC06g33950D	0.00	0.00	0.00	2.38	0.00	0.00	0.00	0.08
BnaCnn.NF-YA3	BnaCnng68450D	0.28	6.23	0.70	0.94	4.18	50.00	10.53	5.31
BnaA05.NF-YA4	BnaA05g09110D	28.47	51.66	27.77	18.87	13.82	25.47	15.71	11.57
BnaC04.NF-YA4	BnaC04g10470D	33.27	62.13	42.52	31.95	8.25	15.39	9.91	8.45
BnaA06.NF-YA5	BnaA06g01280D	0.31	5.87	0.94	0.58	0.38	1.32	0.42	0.28
BnaA08.NF-YA5	BnaA08g00840D	0.17	0.58	0.15	0.02	0.32	0.77	0.31	0.25

BnaAnn.NF-YA5	BnaAnng31560D	0.06	2.36	0.72	0.16	0.75	0.57	1.03	0.30
BnaC06.NF-YA5a	BnaC06g10580D	1.03	5.07	1.84	1.57	2.29	4.18	2.88	1.47
BnaC06.NF-YA5b	BnaC06g06950D	0.64	5.52	1.05	0.92	0.49	2.30	1.35	0.85
BnaCnn.NF-YA5	BnaCnng30790D	0.84	1.21	0.60	0.67	0.73	0.94	1.19	0.69
BnaA01.NF-YA6	BnaA01g29650D	0.00	0.12	0.00	0.00	0.03	0.39	0.09	0.00
BnaC01.NF-YA6	BnaC01g37260D	0.99	36.71	2.33	1.76	5.95	90.87	10.69	7.29
BnaCnn.NF-YA6	BnaCnng46640D	0.00	0.00	0.00	0.00	0.00	0.00	0.00	0.00
BnaA09.NF-YA7	BnaA09g26040D	5.55	19.41	47.02	8.07	4.17	8.17	20.08	2.92
BnaC05.NF-YA7	BnaC05g23480D	0.61	9.35	8.45	1.45	7.85	12.79	18.11	5.13
BnaA09.NF-YA8	BnaA09g44920D	0.00	0.04	0.00	0.02	0.88	0.86	0.89	1.49
BnaA01.NF-YA9	BnaA01g25500D	0.00	0.04	0.07	0.11	0.00	0.00	0.00	0.11
BnaA03.NF-YA9	BnaA03g35980D	0.73	5.62	1.23	0.47	4.46	5.49	4.23	3.24
BnaA05.NF-YA9	BnaA05g19990D	0.00	0.09	0.00	0.00	0.00	0.08	0.00	0.00
BnaC03.NF-YA9	BnaC03g41820D	17.87	25.51	14.93	12.84	16.14	17.93	15.29	11.17
BnaC05.NF-YA9	BnaC05g31370D	4.25	12.49	5.51	4.38	4.59	11.84	7.67	5.23
BnaA10.NF-YA10	BnaA10g24470D	0.45	4.50	0.17	0.05	0.11	15.00	0.38	0.11
BnaC09.NF-YA10	BnaC09g49050D	0.57	12.31	1.08	0.26	0.71	38.50	3.12	1.35
BnaA03.NF-YB1	BnaA03g18200D	0.28	1.04	0.57	0.33	0.63	0.13	0.56	0.52
BnaA05.NF-YB1	BnaA05g06260D	4.20	15.29	6.59	5.41	15.87	18.21	16.28	14.73
BnaC03.NF-YB1	BnaC03g21730D	0.24	1.34	0.40	0.40	0.16	0.25	0.29	0.16
BnaC04.NF-YB1	BnaC04g06490D	2.41	7.42	3.40	3.63	5.74	5.37	5.95	4.06
BnaA02.NF-YB2	BnaA02g25600D	37.87	54.30	38.80	32.88	11.68	11.42	10.50	11.70
BnaA09.NF-YB2	BnaA09g18400D	44.97	53.46	35.97	42.97	11.85	16.61	10.32	9.66
BnaC02.NF-YB2	BnaC02g33400D	14.24	40.53	26.63	12.47	9.81	19.98	16.58	9.65
BnaC07.NF-YB2	BnaC07g20390D	0.00	0.00	0.00	0.00	0.00	0.00	0.00	0.00
BnaC09.NF-YB2	BnaC09g20320D	17.39	18.62	15.73	16.44	8.04	8.11	6.27	9.21

BnaA01.NF-YB3	BnaA01g28350D	17.22	16.87	13.05	12.22	7.33	8.20	9.24	5.24
BnaA03.NF-YB3	BnaA03g33970D	18.10	17.42	17.84	15.06	6.96	5.42	3.59	3.74
BnaC01.NF-YB3	BnaC01g35600D	15.92	16.31	11.34	10.92	14.67	10.72	9.51	7.18
BnaC03.NF-YB3	BnaC03g39380D	23.37	18.70	18.37	19.45	4.40	5.09	4.21	4.35
BnaC05.NF-YB3	BnaC05g37390D	34.89	23.00	27.37	30.36	14.44	16.98	16.35	10.66
BnaC05.NF-YB4	BnaC05g06570D	0.00	0.00	0.00	0.00	2.18	0.71	3.97	4.03
BnaA03.NF-YB5	BnaA03g21860D	0.00	0.00	0.17	0.00	16.63	20.37	22.17	19.92
BnaA05.NF-YB5	BnaA05g00200D	0.00	0.00	0.00	0.00	0.07	0.07	0.39	0.22
BnaC04.NF-YB5	BnaC04g00090D	0.07	0.45	0.08	0.03	0.09	0.25	0.00	0.05
BnaA06.NF-YB6	BnaA06g35340D	0.08	0.05	0.15	0.19	0.09	0.04	0.05	0.14
BnaA09.NF-YB6	BnaA09g18420D	0.50	1.32	0.53	0.83	0.09	0.15	0.05	0.19
BnaC07.NF-YB6	BnaC07g20410D	1.51	2.41	1.36	0.75	1.75	2.30	1.94	2.30
BnaC09.NF-YB6	BnaC09g20350D	0.04	0.12	0.08	0.12	0.13	0.31	0.24	0.09
BnaA03.NF-YB7	BnaA03g38390D	0.00	0.00	0.00	0.00	0.00	0.00	0.00	0.00
BnaA09.NF-YB7	BnaA09g08330D	0.00	0.00	0.00	0.00	0.00	0.00	0.00	0.00
BnaC03.NF-YB7	BnaC03g45220D	0.00	0.00	0.00	0.00	0.00	0.00	0.00	0.00
BnaC09.NF-YB7	BnaC09g08600D	0.00	0.00	0.00	0.00	0.00	0.00	0.00	0.00
BnaA03.NF-YB8	BnaA03g16960D	5.68	7.82	3.00	3.28	20.57	21.00	19.94	17.64
BnaA04.NF-YB8a	BnaA04g21270D	0.57	0.08	0.11	0.22	5.69	3.66	3.00	5.45
BnaA04.NF-YB8b	BnaA04g21250D	0.00	0.00	0.00	0.00	0.00	0.00	0.00	0.00
BnaC03.NF-YB8	BnaC03g20500D	6.46	11.49	3.06	3.90	15.51	19.32	16.04	13.94
BnaCnn.NF-YB8	BnaCnng76740D	0.00	0.00	0.00	0.00	0.00	0.00	0.00	0.00
BnaA07.NF-YB9	BnaA07g10770D	0.00	0.00	0.00	0.00	0.00	0.03	0.00	0.00
BnaAnn.NF-YB9	BnaAnng04140D	0.07	0.34	0.00	0.00	0.52	0.80	0.38	0.48
BnaC07.NF-YB9	BnaC07g14150D	0.91	0.03	0.00	0.60	0.00	0.03	0.00	0.00
BnaCnn.NF-YB9	BnaCnng59920D	0.00	0.00	0.00	0.00	0.00	0.00	0.00	0.00

BnaA09.NF-YB10	BnaA09g33640D	4.12	8.16	5.34	4.24	17.75	24.24	31.82	24.55
BnaC08.NF-YB10	BnaC08g24430D	1.89	4.68	7.09	1.59	12.43	20.20	21.42	19.30
BnaA07.NF-YB11	BnaA07g13230D	4.16	5.45	2.55	3.82	9.33	8.29	9.68	9.41
BnaC03.NF-YB11	BnaC03g47130D	4.63	4.64	4.05	4.50	6.70	5.16	6.51	6.28
BnaC04.NF-YB11	BnaC04g16850D	9.48	10.69	7.56	8.31	17.24	18.29	20.93	20.88
BnaA10.NF-YB12	BnaA10g23160D	2.66	9.76	3.22	3.62	10.22	14.34	11.60	9.67
BnaAnn.NF-YB12	BnaAnng06230D	0.03	0.06	0.00	0.00	0.31	0.24	0.12	0.28
BnaC02.NF-YB12	BnaC02g01260D	1.73	3.90	2.06	1.85	7.79	8.63	7.79	8.38
BnaC09.NF-YB12	BnaC09g47720D	4.82	14.08	4.37	4.46	18.51	16.78	17.26	16.65
BnaA09.NF-YB13	BnaA09g05430D	2.54	7.93	3.48	3.16	10.01	7.90	7.23	7.11
BnaC09.NF-YB13	BnaC09g05000D	3.89	8.69	5.14	4.45	10.73	8.92	9.14	7.71
BnaA06.NF-YC4	BnaA06g16420D	14.69	2.96	5.14	10.66	0.96	0.35	0.32	0.26
BnaAnn.NF-YC4	BnaAnng22700D	34.98	25.03	32.45	36.16	37.83	34.95	37.58	30.11
BnaC03.NF-YC4	BnaC03g50810D	33.32	20.03	38.54	34.95	30.25	22.53	29.88	27.92
BnaC08.NF-YC4	BnaC08g20340D	32.60	10.60	17.66	31.02	5.87	2.94	3.27	3.57
BnaCnn.NF-YC4	BnaCnng15880D	1.58	3.19	1.87	2.24	3.94	4.11	2.99	4.38
BnaA06.NF-YC9	BnaA06g05230D	1.09	17.30	2.16	1.19	1.81	12.17	3.55	2.88
BnaA08.NF-YC9	BnaA08g26690D	0.19	1.69	0.51	0.36	4.76	11.45	9.59	4.65
BnaA09.NF-YC9	BnaA09g48620D	11.06	15.51	7.63	9.94	18.98	23.43	17.14	20.50
BnaC05.NF-YC9	BnaC05g06520D	3.11	20.14	3.47	2.38	1.34	3.25	1.78	1.42
BnaC08.NF-YC9	BnaC08g42880D	20.84	54.77	19.77	21.23	50.07	54.97	48.24	53.57
BnaCnn.NF-YC9	BnaCnng06510D	0.07	0.07	0.47	0.05	0.27	0.84	1.24	0.26
BnaA06.NF-YC10	BnaA06g04450D	2.88	9.01	2.51	3.69	5.67	5.43	4.86	4.12
BnaA09.NF-YC10	BnaA09g49120D	1.49	0.56	1.83	2.19	2.26	1.66	2.02	1.44
BnaC05.NF-YC10	BnaC05g05650D	6.51	9.12	4.75	8.40	12.42	10.00	10.57	10.19
BnaC08.NF-YC10	BnaC08g43430D	1.14	1.46	0.87	1.07	2.70	1.49	1.53	1.01

BnaA01.NF-YC11	BnaA01g30580D	4.99	8.51	3.70	4.14	10.83	11.36	11.37	9.18
BnaA03.NF-YC11	BnaA03g32260D	5.86	11.13	5.11	6.33	7.23	6.18	5.15	5.57
BnaA10.NF-YC11	BnaA10g23530D	4.94	9.09	5.10	4.97	9.74	9.82	10.27	9.20
BnaC01.NF-YC11	BnaC01g38370D	4.80	13.12	4.76	4.58	14.99	16.88	13.60	11.61
BnaC03.NF-YC11a	BnaC03g37550D	4.48	9.59	4.75	5.29	8.59	9.17	8.74	7.07
BnaC03.NF-YC11b	BnaC03g37560D	96.91	2.90	224.92	188.01	30.01	0.62	21.12	36.09
BnaC09.NF-YC11	BnaC09g48240D	9.81	17.90	10.62	8.92	23.07	27.02	18.41	19.23
BnaA06.NF-YC13	BnaA06g36810D	5.23	14.79	5.22	8.75	14.65	11.05	13.01	14.23
BnaC07.NF-YC13	BnaC07g16980D	24.04	33.03	16.27	27.00	24.77	28.32	29.64	37.42

Note: CK, full strength Afdaling's solution; LN, nitrogen deficiency; LP, phosphorus deficiency; LK, potassium deficiency

Table S6. Primers used for quantitative real-time PCR in this study

Gene name	Forward primer	Reverse primer
BnaA01.NF-YA6	GCTTCTTGGCCTCTACATGG	GGCATTGACGAAGATTGGTT
BnaA01.NF-YC11	CGAGATGGAACTCGAGACCG	TGCCTAACTCCATAGGCCAG
BnaA02.NF-YA3	AGTCCAACAGGAAAGCGAAA	ATTGGGAAACCTGAAAACTGG
BnaA03.NF-YA2	GAAGGTCTTCCCTCTGCTCCA	TGGCACAACTCTTGGACTCT
BnaA07.NF-YA3	AAGCTTGAAGGTGGTGGATG	TGTCATTGCGCCTGACTTAG
BnaA09.NF-YC9	ATTGTTCCGAGGGAGGATCT	CTGATCAGGTCCCTGTTGCT
BnaA10.NF-YC11	CGGTATCACGATGCCTTCAC	ACCCGGATACTCCTCTGGTT
BnaC01.NF-YC11	CAGTCCCAGCTGCAGACAA	TCCTCGTCTATCCTCTTACCCA
BnaC05.NF-YA2b	CTGAAACGGTTGTAAGAGCATCTA	GCATATGAACAACACACGATC
BnaC05.NF-YC9	CTTGACACGACACCCACCTTT	CATGGCTCCTGACTGTCCAT
BnaC06.NF-YA3a	TTGCTCCTCCGCAGACTAAT	CATGGTATTGCTTCGCATTG
BnaC08.NF-YC4	GTCCTACCAACGCCAAGAGA	GGTGAGCTCGAGGATGAA
BnaC08.NF-YC9	TTCCGAGGGAGGATCTGAGG	AAGCTAATTCTCCACCAGGGTT
BnaC09.NF-YB12	TGAACAGCGGAGCAGAGATG	AGTTGCTGCTCTTGATCTGGT
BnaC09.NF-YC11	TGCAAGCTGACGAGGATGTT	CATTACCGAGACCGTGACCG
BnaCnn.NF-YA3	AAAGGCCAAGAGGATCTGGT	ATATCAGCACCGTCGGAAAC
EF1-α	GCCTGGTATGGTTGTGACCT	GAAGTTAGCAGCACCCTTGG
